# Clonal raider ant brain transcriptomics identifies candidate molecular mechanisms for reproductive division of labor

**DOI:** 10.1186/s12915-018-0558-8

**Published:** 2018-08-13

**Authors:** Romain Libbrecht, Peter R. Oxley, Daniel J. C. Kronauer

**Affiliations:** 1 Laboratory of Social Evolution and Behavior, The Rockefeller University, 1230 York Avenue, New York, NY 10065 USA; 2 Institute of Organismic and Molecular Evolution, Johannes Gutenberg University, Johannes-von-Müller-Weg 6, 55128 Mainz, Germany; 3 Samuel J. Wood Library, Weill Cornell Medicine, 1300 York Avenue, New York, NY 10065 USA

**Keywords:** Eusociality, Social behavior, Social insects, Gene expression, Gene regulation, Time course, Brood care, Reproduction

## Abstract

**Background:**

Division of labor between reproductive queens and workers that perform brood care is a hallmark of insect societies. However, studies of the molecular basis of this fundamental dichotomy are limited by the fact that the caste of an individual cannot typically be experimentally manipulated at the adult stage. Here we take advantage of the unique biology of the clonal raider ant, *Ooceraea biroi*, to study brain gene expression dynamics during experimentally induced transitions between reproductive and brood care behavior.

**Results:**

Introducing larvae that inhibit reproduction and induce brood care behavior causes much faster changes in adult gene expression than removing larvae. In addition, the general patterns of gene expression differ depending on whether ants transition from reproduction to brood care or vice versa, indicating that gene expression changes between phases are cyclic rather than pendular. Finally, we identify genes that could play upstream roles in regulating reproduction and behavior because they show large and early expression changes in one or both transitions.

**Conclusions:**

Our analyses reveal that the nature and timing of gene expression changes differ substantially depending on the direction of the transition, and identify a suite of promising candidate molecular regulators of reproductive division of labor that can now be characterized further in both social and solitary animal models. This study contributes to understanding the molecular regulation of reproduction and behavior, as well as the organization and evolution of insect societies.

**Electronic supplementary material:**

The online version of this article (10.1186/s12915-018-0558-8) contains supplementary material, which is available to authorized users.

## Background

The evolution of social life from solitary organisms, one of the major transitions in evolution [1], is best exemplified by eusocial hymenopterans (ants, some bees, and some wasps). At the core of hymenopteran societies lies reproductive division of labor, whereby one or several queens monopolize reproduction while workers perform all the non-reproductive tasks necessary to maintain the colony [2]. To better understand the evolution of eusociality requires investigating the mechanisms that plastically regulate reproductive and non-reproductive tasks in social insects.

Studies of reproductive division of labor have primarily focused on comparing the queen and worker castes, both at the adult stage and during larval development when caste differentiation occurs [3–13]. Such studies have provided valuable insights into the mechanisms regulating the alternative developmental trajectories of queens and workers, and contributed greatly to the elaboration of theories regarding the evolution of eusociality [14–19].

However, there are three major limitations associated with the comparison of morphologically distinct queens and workers. First, at the adult stage, the two castes not only differ in reproductive status and behavior, but also in morphology, baseline physiology, immunity, and lifespan [2, 20, 21]. Thus it is difficult to disentangle differences between queens and workers that are actually associated with plastic variation in reproduction and behavior from those associated with other traits. Second, the caste is fixed when females reach adulthood and thus cannot be experimentally manipulated in adults, making it challenging to establish causality between molecular and phenotypic differences. Third, morphologically distinct queen and worker castes represent the derived state: comparing them does not necessarily provide accurate information on the mechanisms under selection during the evolutionary transition to eusociality from a totipotent ancestor. These limitations do not apply to eusocial insect species with flexible queen and worker castes, and studying the molecular basis of reproductive division of labor in such species has the potential to provide complementary insights to studies of species with fixed morphological castes [22–24].

Eusocial hymenopterans are derived from subsocial wasp-like ancestors that alternated between reproductive and brood care phases [15, 25–27]. The evolution of eusociality involved a decoupling of these phases in different individuals, the queens and the workers, respectively. To understand the evolution of such decoupling requires investigating the molecular mechanisms regulating the transitions between phases. Unfortunately, extant wasp species with a subsocial cycle and progressive provisioning of their larvae are rare tropical species (e.g., *Synagris* wasps in sub-Saharan Africa [28] or *Stenogaster* wasps in southeast Asia [29]) that have not been studied from a molecular perspective because they cannot be experimentally manipulated under controlled laboratory conditions.

**Fig. 1:**
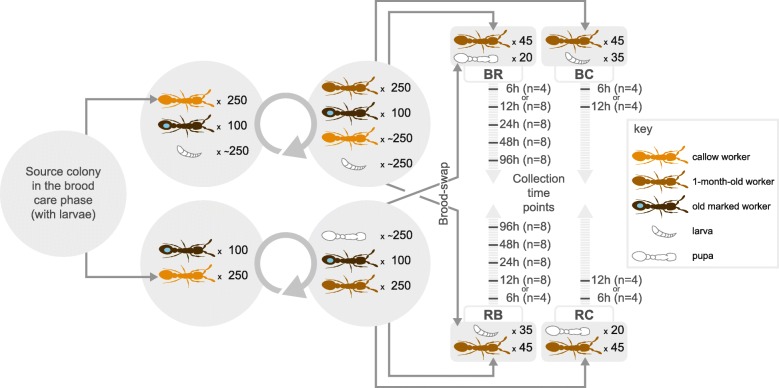
Design of the brood-swap experiment. For each biological replicate, a large source colony in the brood care phase was used to establish two colonies of 250 1-month-old workers and 100 marked ≥ 3-month-old workers. One of these colonies received approximately 250 larvae. After a full colony cycle, each colony contained a complete cohort of brood and workers and was in either peak brood care phase (with larvae) or early reproductive phase (with eggs and pupae). On the day the first eggs were laid, the 1-month-old workers were subdivided in colonies of 45 workers each. One colony from each phase served as the control colony and was given brood from the mother colony. The remaining colonies received brood from the mother colony in the opposite phase of the cycle, triggering the transition toward the alternative phase. Colonies were subsequently collected 6, 12, 24, 48, or 96 h post treatment. BR workers transitioning from the brood care phase to the reproductive phase (after larvae were removed and pupae added), RB workers transitioning from the reproductive phase to the brood care phase (after pupae and eggs were removed and larvae added), BC workers from the brood care phase with larvae (brood care phase control), RC workers from the reproductive phase with pupae (reproductive phase control)

The clonal raider ant *Ooceraea biroi* (formerly *Cerapachys biroi* [30]) is a promising model system to study the evolution of eusociality because it alternates between reproductive and brood care phases in a cycle that is reminiscent of the subsocial cycle of the ancestors of eusocial hymenopterans [31, 32]. This species has no queen caste, and colonies consist of morphologically uniform and genetically identical workers. Colonies alternate between reproductive phases of ca. 18 days during which workers reproduce asexually in synchrony and brood care phases of ca. 16 days during which workers have regressed ovaries, forage, and nurse larvae [31, 33]. Social cues derived from the larvae regulate the transitions between phases: when larvae hatch towards the end of the reproductive phase, they soon suppress ovarian activity and induce brood care behavior in the adults, and when larvae pupate towards the end of the brood care phase, the adults begin to activate their ovaries and foraging activity ceases [34, 35]. This allows precise experimental manipulation of the cycle by adding or removing larvae of a particular developmental stage at standardized time points during the cycle (Fig. 1). At the same time, *O*. *biroi* affords maximal control over the genetic composition and age structure of experimental colonies, arguably the two most important factors that affect division of labor in social insects [31, 35–37]. This study takes advantage of the unique biology of *O*. *biroi* to investigate the molecular underpinnings of behavioral transitions from reproduction to brood care and vice versa, and identify candidate genes potentially involved in the evolutionary transition from subsocial to eusocial living.

## Results

We experimentally manipulated the presence of larvae in *O*. *biroi* colonies of age-matched, genetically identical individuals to induce transitions from the reproductive to the brood care phase (hereafter “RB transition”) or from the brood care to the reproductive phase (hereafter “BR transition”). We then collected brain gene expression data from individuals sampled across five consecutive time points at 6, 12, 24, 48, and 96 h post manipulation (eight biological replicates per time point) to evaluate gene expression changes over time in response to changes in brood stimuli (Fig. 1). After checking for outliers, we judged the 6-h time points to mostly reflect a response to recent experimental disturbance and thus removed them from further analysis (“Methods”; Additional file 1).

### Brain gene expression changes when ants transition between phases

We conducted two independent differential expression analyses (one for each transition) that revealed 2043 genes with significant changes in expression over time in the RB transition (hereafter “RB-DEGs”) and 626 genes with significant changes in expression over time in the BR transition (hereafter “BR-DEGs”) (adjusted *p* values < 0.05; “Methods”). These analyses also detected genes with similar changes in expression over time in both transitions, which likely stem from experimental manipulations. Thus we conducted a more conservative analysis that would not detect such genes by identifying genes that showed transition-specific expression changes over time (“Methods”). We detected 596 genes with different changes in expression over time between RB and BR transitions (hereafter “DEGs”; time-by-transition interaction with adjusted *p* values < 0.05; gene identifiers and annotations in Additional file 2).

**Fig. 2:**
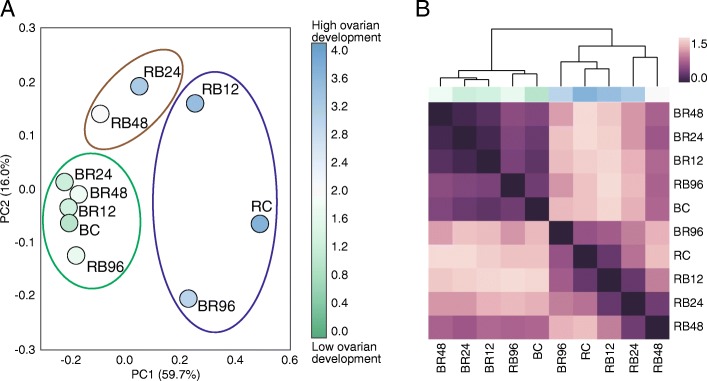
Cluster analysis of samples based on the mean gene expression of each time point, for 596 differentially expressed genes (adjusted *p* value ≤ 0.05). **a** PCA plot of brood-swap and control samples. Percentages on each axis indicate the proportion of variance explained by the indicated principal component. The blue, brown, and green ellipses show the k-means cluster assignment. The color of each sample indicates the average ovary activation score as per [77]; 0 indicates no signs of ovary activation while 4 indicates fully developed eggs are present. Sample names are as per Fig. 1. **b** Heatmap showing Euclidean distances between all time points. The dendrogram was constructed using the average distances between time points. The blue and green color bar above the heatmap indicates average ovary activation score, as in **a**. Sample names are as per Fig. 1

PCA clustering of samples according to brain gene expression segregated samples according to ovary score (Fig. 2a). Samples that were early in the transition were most similar to their corresponding control samples. Samples that were late in the transition were most similar to the control samples for the opposite transition (i.e., closest to the phase opposite from where they started in the experiment). This shows that our experimental timeline appropriately spanned both transitions from beginning to end and that brain gene expression is an accurate corollary of the ovarian development of *O*. *biroi* individuals.

### The timing of gene expression changes differs between transitions

The average distance between samples (Fig. 2a, b) indicated a more gradual change in gene expression when transitioning to the brood care phase than when transitioning to the reproductive phase. The unbiased clustering of samples further suggested that changes in gene expression patterns occurred earlier after adding larvae to ants in the reproductive phase than after removing larvae from ants in the brood care phase (Fig. 2a). Only samples collected 12 h after addition of larvae clustered with the control samples for the reproductive phase, while later samples clustered either as an intermediary group (24 and 48 h) or with the brood care phase controls (96 h) (Fig. 2a). On the other hand, following the removal of larvae, all samples collected before 96 h clustered with the control for the brood care phase (Fig. 2a).

**Fig. 3:**
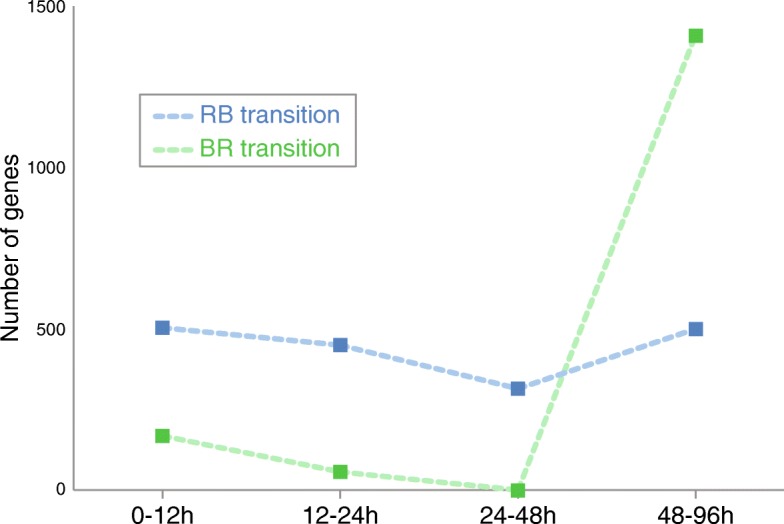
Number of genes in clusters (enriched for DEGs) with maximal change in expression for each time interval. The distribution of such numbers across time intervals differed significantly between transitions (*χ*^2^ = 1217.5, *p* < 0.00001, Additional file 5). This suggests that the transition from reproduction to brood care (RB transition; blue) and the transition from brood care to reproduction (BR transition; green) are associated with distinct time dynamics of gene expression

To further test whether gene expression dynamics differed between transitions, we used P-spline smoothing with mixed effects models [38] to fit the gene expression time course profiles into clusters (i.e., groups of co-expressed genes over time). This approach grouped all genes into 76 clusters for the BR transition and 96 clusters for the RB transition (Additional files 3 and 4). In order to compare clusters, we also identified their “maximum change vector” (MCV), which is the interval, magnitude, and direction of the largest average gene expression change between time intervals. For each transition, we used the MCV values to determine the number of genes showing their maximum change in expression for each time interval. If the timing of gene expression changes was similar in both transitions, we would expect a comparable distribution of such number of genes across time intervals for clusters showing significant changes over time (i.e., clusters enriched for DEGs). Contrary to this expectation, we found that among clusters enriched for DEGs, the distribution differed significantly between transitions (*χ*^2^ = 1217.5, *p* < 0.00001, Fig. 3, Additional file 5). Consistent with the PCA analysis, most gene expression changes in the BR transition occurred between 48 and 96 h, whereas changes in the RB transition were weighted towards earlier time intervals (Fig. 3).

### The nature of gene expression changes differs between transitions

The independent analyses of the RB and BR transitions revealed a weak overlap between the lists of RB-DEGs and BR-DEGs: only 7.4% (185/2484) of the genes differentially expressed over time in one transition were differentially expressed over time in both transitions. In addition, 55.7% (103/185) of the overlapping DEGs had the same MCV in both transitions, suggesting that their expression changes were a result of experimental manipulation. This suggests that the genes and/or pathways associated with transitioning between phases are specific to each transition.

The gene co-expression clusters further corroborate this finding. Constructing a network from cluster membership in both transitions revealed a highly connected, homogenous network (Additional file 6), showing that most genes were co-expressed with different genes in each transition. This is similarly illustrated by cluster enrichment for Gene Ontology (GO) terms. We found 27 enriched clusters (including four clusters enriched for DEGs) for the BR transition and 35 (including seven clusters enriched for DEGs) for the RB transition (Additional file 3). Among clusters enriched for DEGs, only 6.9% (2/29) of the GO terms associated with one transition were also associated with the other transition (Additional file 7).

Furthermore, the expression patterns of genes that were co-expressed with the same genes in both transitions were inconsistent with a symmetrical molecular regulation. We identified all conserved co-expression clusters in the network (i.e., clusters whose members were more similar between transitions than expected by chance) (“Methods”, Additional file 6). If the primary molecular mechanisms regulating phase transitions were reversible, then co-expressed genes would show expression changes in opposite directions in each transition. In that case, network edges that link clusters of genes regulated in opposite directions between transitions would represent a higher proportion of edges in the conserved network (with only non-random connections) compared to the complete network (which includes random connections). We found the reverse pattern: edges linking clusters of genes showing opposite changes of expression over time between transitions were less frequent in the conserved network (24.4%) than in the complete network (45.8%; *χ*^2^ = 22.4, *p* < 0.00001; Additional file 8).

### Using the time course data to identify candidate genes

**Fig. 4:**
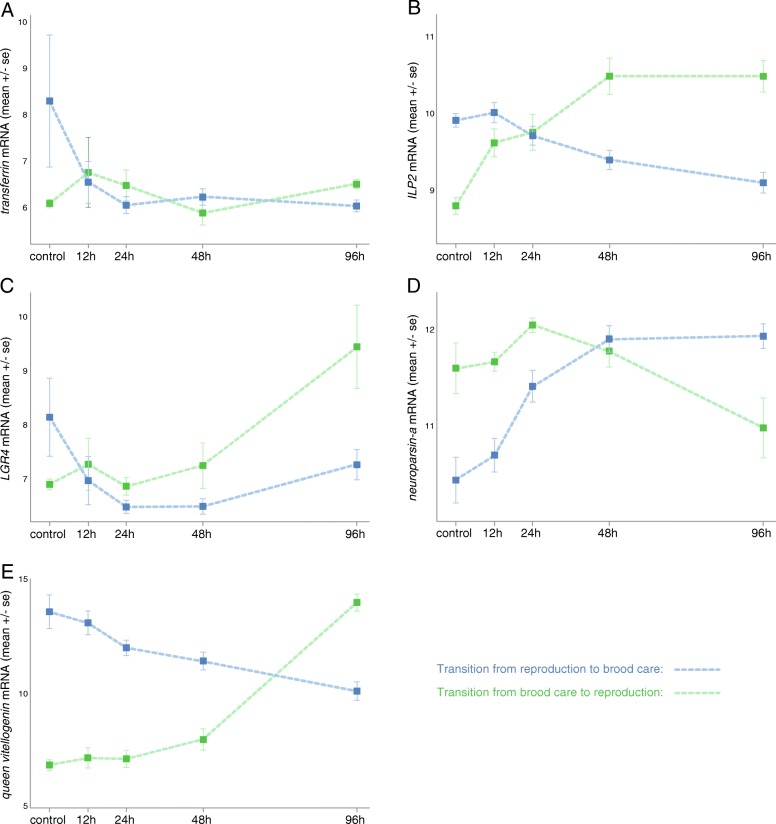
Select candidate genes for the regulation of the transitions between brood care and reproduction. The plots show the expression changes over time after adding larvae (RB transition; blue) or removing larvae (BR transition; green) from the colonies. **a** *Transferrin*. **b** *ILP2* (*insulin-like peptide 2*). **c** *LGR4* (*leucine-rich repeat-containing G-protein-coupled receptor 4*). **d** *Neuroparsin-a*. **e** *Queen vitellogenin*. Gene expression is shown as variance-stabilized transformed read counts (which approximate log2-transformed read counts)

Ranking the 596 DEGs according to their change in expression between the control and the 96-h time point for each transition allowed us to identify genes most likely to be involved in the molecular regulation of one or both transitions (the lists of the top 40 DEGs when ranked according to log2 fold change are available in Additional file 9). This includes genes that encode proteins with neuroendocrine functions (*queen vitellogenin*), neuropeptides (*insulin-like peptide 2*, *neuroparsin-A*), and neuropeptide receptors (*leucine-rich repeat-containing G-protein-coupled receptor 4*) and enzymes involved in neuropeptide processing (*carboxypeptidase M*, *aminopeptidase N*), neurotransmitter receptors (*glycine receptor subunit alpha 2*) and proteins involved in neurotransmission (*synaptic vesicle glycoprotein 2C*, three *kinesin-like proteins*), neuronal function (*leucine-rich repeat neuronal protein 2*, *trypsin inhibitor*, *gliomedin*), hormone binding (*transferrin*), transcription (*hunchback*, *transcription termination factor 2*, *speckle-type POZ protein B*, *zinc finger BED domain-containing protein 1*, *lymphoid-specific helicase*), and protein synthesis and modification (*peptidyl-prolyl cis-trans isomerase D*, *hyaluronan-mediated motility receptor*, *alpha-(1,3)-fucosyltransferase 6*). The expression patterns for some of these candidate genes are shown in Fig. 4. In addition, we identified among these genes those with highest change in expression between the control and the 12-h time point (Additional file 9), i.e., genes that could function upstream in the molecular processes regulating the transitions. These include candidate genes with early changes in the RB transition (*hunchback*, *alpha-(1,3)-fucosyltransferase 6*), in the BR transition (*insulin-like peptide 2*, *glycine receptor subunit alpha 2*, *transcription termination factor 2*, *hyaluronan-mediated motility receptor*, *annulin*), or in both transitions (*leucine-rich repeat-containing G-protein-coupled receptor 4*, *leucine-rich repeat neuronal protein 2*, *transferrin*).

### Both transitions are associated with overlapping sets of transcription factors

For each transition, we tested whether gene clusters were enriched for transcription factor binding sites. We used the JASPAR database to identify 27 clusters (including four clusters enriched for DEGs) in the BR transition and 12 clusters (including four clusters enriched for DEGs) in the RB transition that were significantly enriched for transcription factor binding sites (Additional file 3). A number of transcription factors were repeatedly associated with clusters enriched for DEGs and were present in both transitions (Additional file 10). Of particular note, in each transition, there was only one cluster enriched for a single transcription factor binding site, and in both cases, it was for the *forkhead* binding site. We identified all genes with at least one highly conserved binding site for *forkhead* (“Methods”) to show that these genes cluster samples according to ovary activation and chronological distance (Additional file 11), which is consistent with *forkhead* being involved in the regulation of both transitions.

## Discussion

Colonies of *O*. *biroi* alternate between brood care and reproductive phases, and our time course analyses of the brain transcriptome reveal that the transitions from brood care to reproduction and from reproduction to brood care involve distinct overall patterns of gene expression changes. The timing of brain gene expression changes after manipulating social cues differs between transitions. The addition of larvae leads to a rapid change in gene expression, whereas larval removal results in a much slower change. Inappropriately timed production of eggs incurs individual and colony-level fitness costs. At the individual level, eggs laid in the presence of larvae are eaten, wasting the resources taken to produce them. Furthermore, individuals with active ovaries are aggressed and eventually killed by nestmates [35]. Such policing behavior has been hypothesized to minimize colony-level costs because unsynchronized egg-laying would jeopardize the colony cycle [39]. Such fitness costs will exert selective pressure on the regulation of reproductive physiology [40]: in line with our findings, regulatory mechanisms should be slow to activate ovaries and quick to suppress or reverse egg production.

In addition, our results are consistent with larval cues acting as a reinforcement signal for brood care and for inhibition of reproduction, because the removal of the brood signal is accompanied by a delay in gene expression and physiological adjustments. Such a delay is necessary in *O*. *biroi* to prevent premature transitioning to reproduction, such as during foraging, when some individuals frequently exit the nest during the brood care phase and are thus only sporadically exposed to larval cues. Comparable resistance to change has been observed in other species and contexts. In behavioral sciences, the resistance to change in behavior after removal of a stimulus has been compared to the inertial mass [41] and applied to behaviors as diverse as drug addiction in humans [41] or food-reinforced behaviors in birds [42]. Physiological regulations are also subject to resistance to change. For example, physiological changes that occur in rats in response to a stressful stimulus (e.g., cold temperature or low oxygen) take several days to return to baseline levels after stimulus removal [43, 44]. This pattern of rapid response to stimulus exposure but slow response to stimulus removal also parallels the adaptation and deadaptation rates seen in many molecular systems, such as the cAMP-mediated cGMP response inducing cell aggregation in the slime mold *Dictyostelium discoideum* [45].

Our findings are not consistent with the *O*. *biroi* colony cycle being regulated by discrete gene networks in which expression is coordinately and symmetrically up- or downregulated during transitions between phases. Indeed, neither the differential expression nor the network analyses found substantial overlap in gene membership between transitions. In other words, the sequence of gene expression changes that is associated with the transition to the reproductive phase is not the reverse sequence of gene expression changes associated with the transition to the brood care phase.

Finding transcriptome-wide differences in expression between transitions does not necessarily imply that individual genes or groups of genes cannot play a regulatory role in both transitions. In fact, some of the candidate genes identified here are involved in both the BR and the RB transition (see below). Our differential expression analysis revealed that genes with some of the highest expression changes over time have neuroendocrine, neuronal, and gene regulatory functions, and regulate neuropeptide signaling and neurotransmission. Among these genes, we have highlighted five candidates for the regulation of reproduction and brood care in *O*. *biroi* (Fig. 4) by identifying the DEGs with the largest changes in expression along one or both transitions (Additional file 9) that belong to molecular pathways with caste-biased activity in other social insects.

The gene *transferrin* (Fig. 4a) has large and early changes in expression in both transitions and shows caste-biased expression in multiple species of social insects. In the ant *Temnothorax longispinosus* and in the wasp *Polistes canadensis*, whole-body RNA sequencing revealed higher expression in queens compared to workers [5, 46]. While in insects the protein encoded by *transferrin* transports iron into the eggs, reduces oxidative stress, and interacts with the vitellogenin and juvenile hormone pathways [47], its role in the brain remains poorly understood.

Another candidate gene identified in our study is *insulin-like peptide 2* (*ILP2*; Fig. 4b), a neuropeptide that belongs to the insulin signaling pathway, which is a conserved pathway that regulates nutrition, fertility, and longevity in animals [48, 49]. Insulin signaling, together with the juvenile hormone and vitellogenin pathways [50–52], is involved in caste determination and division of labor in social insects [14, 50, 53–56]. Interestingly, *ILP2* shows one of the earliest responses to the removal of larvae (in the BR transition), and it has recently been shown that *ILP2* indeed regulates ant reproduction [57]. Another candidate gene with early expression changes in both transitions is *leucine-rich repeat-containing G-protein-coupled receptor 4* (*LGR4*; Fig. 4c). It encodes a G-protein-coupled receptor predicted to bind relaxin-like peptides [58], which belong to the insulin family, together with insulin-like peptides and insulin-like growth factors [59]. The expression of the neuropeptide *neuroparsin-a* increases gradually when transitioning to brood care (Fig. 4d), which is consistent with neuroparsins having anti-gonadotropic roles through interactions with the vitellogenin and insulin signaling pathways [60]. Together, these expression patterns support the hypothesis that insulin signaling plays an important role in linking changes in social cues to reproductive changes [23, 57, 61].

*Queen vitellogenin* (Fig. 4e) is differentially expressed between reproductive and non-reproductive castes in multiple species of ants, bees, wasps, and termites [5, 9, 17, 31, 53, 62–66]. This gene encodes the yolk protein precursor vitellogenin, which is instrumental to egg formation. In formicoid ants, the *vitellogenin* gene has been duplicated, and some gene copies have been co-opted to regulate non-reproductive functions such as behavior [17, 67]. The changes in *queen vitellogenin* expression mirror the ovarian development and overall alterations of the transcriptome: *queen vitellogenin* displays a gradual and early decrease during the RB transition but a sharp and delayed increase during the BR transition (Fig. 4e).

The protein vitellogenin is typically synthesized in the fat body, secreted into the hemolymph, and transported into the developing oocytes [68]. In addition, vitellogenin has been localized in the honeybee brain, suggesting that it also has a neuroendocrine function [69]. Here we show that *vitellogenin* gene expression in the brain is correlated with changes in reproductive physiology. This finding is consistent with *vitellogenin* changes in expression associated with adult caste differentiation and reproductive activation in queenless ants of the genus *Diacamma* [23] and with previous reports of caste-biased *vitellogenin* expression in the head [31, 53] or in the brain [64]. Such accumulation of evidence for caste-biased *vitellogenin* expression across the phylogeny of social insects, and in species with and without distinct morphological castes, identifies *vitellogenin* genes as key players in the evolution and regulation of reproductive division of labor. Our analyses of gene expression changes over time reveal that, although *queen vitellogenin* shows one of the highest changes in expression in both transitions, such changes occur rather late after manipulating social cues. This supports the notion that the role of vitellogenin in the brain is likely to be downstream of earlier molecular changes (e.g., in the insulin signaling pathway) [57].

A recent study compared gene expression between reproductive and non-reproductive *Diacamma* ants, where caste is determined at the adult stage via social dominance and aggressive interactions [23]. Similar to *O*. *biroi*, this avoids the problem of morphological differences between castes and allows for the induction of changes in behavior and reproduction by experimentally manipulating social interactions. Interestingly, despite several differences in experimental design, the overlap between the genes differentially expressed in *Diacamma* [23] and *O*. *biroi* includes genes in the insulin signaling and vitellogenin pathways. Given that the two species are phylogenetically only distantly related, this opens the possibility that these genes are important in regulating reproductive division of labor across the ants and may have played a role during the evolutionary origin of ant eusociality [57].

Recent studies have proposed that changes in gene regulatory mechanisms were associated with the evolution of eusociality [70, 71]. In our study, many DEGs that showed early changes in gene expression have gene regulatory functions such as the onset (*hunchback*) and termination (*transcription termination factor* 2) of transcription, as well as the synthesis (*PPID*, *annulin*), glycosylation (*alpha-(1,3)-fucosyltransferase 6*), and phosphorylation (*hyaluronan-mediated motility receptor*) of proteins. In addition, gene clusters enriched for DEGs were also frequently found to be enriched for genes with certain transcription factor binding sites. This suggests complex transition-specific gene expression and regulation, affected by multiple transcription factors. Nevertheless, genes that are putatively regulated by a few transcription factors exhibit predictable patterns of regulation. For example, the expression of genes associated with *forkhead* transcription factor binding sites provided significant predictive power as to the physiological state of an individual. Interestingly, *forkhead* transcription factors regulate reproduction in other insect species. For example, knocking down *forkhead* transcription factors in the yellow fever mosquito *Aedes aegypti* and the brown planthopper *Nilaparvata lugens* reduced offspring production and the activity of the vitellogenin pathway [72, 73]. In addition, *forkhead* plays a role in the regulatory network of salivary glands in insects [74], which include the mandibular glands that produce caste-specific compounds in honeybees [75]. Interestingly, the promoter region of *forkhead* shows a depletion of transcription factor binding sites in ants compared to solitary insects, which may have facilitated *forkhead* pleiotropy and its implication in caste-specific regulatory networks [71]. The decoupling of brood care and reproductive phases in different female castes during the evolution of eusociality was associated with the co-option of gene function and regulation [15]. Our findings suggest that transcription factors such as *forkhead* may be among the regulatory elements responsible for the co-option of gene regulatory networks during this evolutionary transition.

## Conclusion

Assuming that the colony cycle of *O*. *biroi* indeed represents a partial reversal to the life cycle of the subsocial ancestor of ants and possibly other eusocial hymenopterans, one parsimonious way to compartmentalize such a cycle would be to disrupt the transition to brood care in response to larval cues in a subset of individuals, which would then act as queens. Given that these queens would now lay eggs continuously, any additional females that emerge at the nest would immediately and permanently be exposed to larval cues and thus locked in the brood care phase of the ancestral cycle. This would then give rise to reproductive division of labor, which could be acted upon by natural selection, driving continued divergence in fertility, and ultimately leading to eusociality. In this study, we report that patterns of gene expression changes over time differ between the transition to brood care and the transition to reproduction in *O*. *biroi*. Our results are therefore not consistent with the transitions being regulated by mirrored sequences of gene expression changes in a pendular manner. On the contrary, patterns of gene expression appear to be circular, with the involvement of transition-specific sets of genes. This implies that, on a molecular level, the transition to brood care could have been disrupted in a variety of ways without affecting the reverse transition. However, especially given our finding that exposure to larval cues entails rapid and large-scale changes in brain gene expression, we would assume that this disruption happened early and upstream in the gene expression cascade. Our time-course data allowed us to identify molecular candidate pathways that respond rapidly to larval cues and could therefore be upstream of the longer-term behavioral and physiological responses. These constitute prime candidates, both for broad comparative analyses across social hymenopterans and for functional experiments in *O*. *biroi* and other species.

## Methods

### Biological samples

Source colonies (Fig. 1) were derived from two separate clonal lineages: MLL1 and MLL4 [76]. Clonal lineage and source colony identity are recorded for all RNA sequencing libraries, which are uploaded to the NCBI Bioproject PRJNA273874. Large source colonies in the brood care phase were used to establish two experimental colonies each (250 1-month old workers and 100 ≥ 3-month old workers), one of which received approximately 250 larvae. After a full colony cycle, each colony contained a complete cohort of brood and workers and was in either peak brood care phase or early reproductive phase. On the day the first eggs were laid in the reproductive phase colony, the 1-month old workers were subdivided into colonies of 45 workers. One of these colonies from each phase served as the control colony and was given brood from the colony the workers were derived from (i.e., larvae for the brood care phase control and eggs and pupae for the reproductive phase control). The remaining colonies received brood from the colony at the opposite stage of the cycle (sub-colonies originally in the reproductive phase received larvae and vice versa), thereby inducing the transition toward the opposite phase. All colonies with larvae were fed every 24 h, immediately after samples for the respective time points had been collected. Colonies were collected 6, 12, 24, 48, or 96 h after experimental manipulation. This process was repeated eight times: four times with and four times without the 6-h time point. In each instance, the control sample was collected at the same time as the earliest time point. After looking for outliers, we removed all samples collected at the 6-h time point (see details below), thus resulting in four biological replicates for the controls and eight biological replicates per time point in both transitions (Fig. 1, Additional file 12). Source and experimental colonies were kept at 25 °C and 60% humidity, and when in the brood care phase were fed frozen *Solenopsis invicta* brood.

### Sample collection and RNA sequencing

At the specified time for each colony, all ants were flash frozen and subsequently stored at − 80 °C. Ovaries and brains were dissected in 1× PBS at 4 °C. To estimate ovarian development, ovary activation was scored according to [77] for 200 ants (20 ants per time point) from two source colonies (Additional file 13). Brains of individuals with two ovarioles were transferred immediately to Trizol, and once ten brains from a colony were pooled, the sample was frozen on dry ice.

RNA was extracted with RNEasy column purification, as explained in Oxley et al. [31]. Clontech SMARTer low input kits were used for library preparation, and RNA sequencing was performed on a HiSeq 2000, with 100 bp single-end reads. Sequencing batches included all time points for both transitions of any given colony, for two source colonies at a time.

### Identification of outlier samples

Nine hundred sixty-seven genes had more than twofold change in expression across all samples. Because these genes contribute the greatest variation between samples, they were used to observe the general pattern of sample clustering, in order to remove outlier samples prior to differential gene expression analysis (Additional file 1).

All 6-h samples (controls and treatments) clustered more closely with each other than with their respective (expected) transition groups. Looking at individual gene expression time courses, it was clear that the 6-h time points frequently deviated wildly from the other time points. This suggests that the majority of gene expression changes observed in the 6-h time points was induced by the experimental disturbance. However, removing the 6-h time points could prevent us from detecting genes that legitimately changed as a result of the actual brood-swap, instead of the experimental manipulation. We therefore looked at the change in sensitivity and specificity of the experiment after removing the 6-h samples from the analysis.

Removing the 6-h time points reduced the number of genes with greater than or equal to twofold difference by 335. Fifty-one percent of these 335 genes were differentially expressed between 6- and 12-h control samples of the same phase and were therefore a priori likely to be false positives. Seventy-three genes were expressed greater than or equal to twofold between 6-h control and treatment samples and were therefore potentially genes regulated by the change in brood stimuli. Of these 73 genes, only 5 were not present in the 632 genes still identified as having greater than or equal to twofold differences after removal of the 6-h time points (Additional file 1). If these genes were real target genes, we would only lose 6.8% of the early-responding genes. Removing the 6-h time points as outliers therefore increased the specificity of our differential expression analysis, with negligible loss of sensitivity.

### Identification of differentially expressed genes

Fastq reads from all samples were aligned to the *Ooceraea biroi* genome (NCBI assembly CerBir1.0) using STAR (default parameters). HTSeq was then used to determine the number of reads aligned to each gene (NCBI *Cerapachys biroi* Annotation Release 100). DESeq2 was used for differential gene expression analysis.

To analyze each transition separately, we contrasted the following models in DESeq2:

- Full model: colony + bs(time, df = 3)
- Reduced model: colony

using the bs function from the splines library (v. 3.2.3) in R for evaluating the spline function of all time points (controls coded as time 0). This contrast identified genes with a significant change at any point in time, not just genes significantly different from the control samples. This analysis was run for both BR and RB transitions.

To account for the effects of experimental manipulation, the following models were contrasted:

- Full model: colony + transition + bs(time, df = 3) + transition: bs(time, df = 3)
- Reduced model: colony + transition + bs(time, df = 3)

This model contrast identified the genes that were differentially expressed over time, after accounting for the differences in gene expression between reproduction and brood care phases. Without using the spline function, we could simply be comparing gene expression at each time point to “time 0” (i.e., the control samples). However, this would not reveal genes whose expression changed temporarily, before returning to their baseline value.

We identified only those genes with a significant time by transition interaction. It has been shown that expression of certain genes can have opposing effects, depending on the context [78]. Genes that show significant change in expression over time, but no significant interaction with phase, may therefore still be important in regulating transitions between phases. However, such genes are confounded with, and cannot be disentangled from, genes that are expressed as a stress response resulting from the brood-swap experimental procedure, and we therefore decided to ignore them in our present analyses.

### Clustering of gene expression time courses

We clustered the samples using P-spline smoothing and mixed effects models according to the algorithm by Coffey et al. [38]. To determine the optimal number of clusters for each transition, we calculated the BIC score for all even cluster sizes between 2 and 120 clusters (Additional file 4). We selected the smallest cluster size of the lower BIC values that did not precede a higher BIC value (Additional file 4).

### Enrichment analyses for expression clusters

Transcription factor binding site enrichment of each cluster was determined with Pscan, using the available position weight matrices from the JASPAR database. Assessment of clusters for enrichment for DEGs and GO terms was determined using Fisher’s exact test followed by Benjamini and Hochberg [79] false discovery rate adjustments. GO term enrichment was calculated using genomepy’s genematch.py module (github.com/oxpeter/genomepy). To identify all *O*. *biroi* annotated genes with *forkhead* transcription factor binding sites, we used the R packages TFBSTools and biostrings, with the position weight matrix for *Drosophila* from the JASPAR database and a 95% minimum score for matching.

### Network analysis of the identified clusters

We first constructed the complete network that consisted of all gene clusters from both transitions. Each node in this network represented a cluster of genes, and edges represented the genes that are shared between clusters. Since each gene is uniquely assigned to a single cluster in each transition, no two clusters from the same transition will ever be connected. Similarly, every gene is represented once, and only once, among all the edges.

The conserved network was constructed by looking at the Jaccard Index for each pair of clusters as a measure of similarity that does not rely on untested assumptions. We then conducted a permutation analysis by calculating 1000 random cluster networks (each cluster had the same number of genes as the original) and calculated the Jaccard Indices of all node pairs. Our conserved network was then created by choosing only those edges that represent a Jaccard Index greater than 95% of all scores from the random networks.

## Additional files

Additional file 1: Outlier analysis. PCA and distance map of genes showing greater than twofold change in expression. A) PCA plot of brood-swap and control samples. Clustering was based on the mean gene expression of each group, for 967 genes with more than twofold change in expression between samples. Percentages on each axis indicate the proportion of variance explained by the indicated principal component. The color of each sample indicates the expected similarity to the control samples; dark blue indicates reproductive phase and dark green indicates brood care phase. Sample names are as per Fig. 1. B) Heatmap showing Euclidean distance between all samples, based on all genes with more than twofold change in expression, and clustered according to average distances between samples. Blue and green color bar above the heatmap indicates similarity to control samples, as in A. Sample names are as per Fig. 1. C) Venn diagram showing outcome of eliminating the 6-h time points. Numbers in the small circles indicate genes with greater than twofold change in expression between 6-h control and treatment samples in the reproduction to brood care transition (blue) and brood care to reproduction transition (green) (a priori true positives). Red numbers indicate genes that show greater than twofold change in expression after removal of the 6-h time points. Thus, elimination of the 6-h samples does not substantially reduce the number of DEGs identified with large expression changes. (PDF 79 kb)

Additional file 2: All 596 DEGs ranked according to *p* value (smaller to larger). (PDF 101 kb)

Additional file 3: All gene clusters identified, and their corresponding enrichment for differentially expressed genes, gene ontology terms, and transcription factor binding sites. (PDF 124 kb)

Additional file 4: Evaluation of BIC scores for selection of optimal number of clusters. Genes were clustered into all even numbered cluster sizes between 2 and 120 (brood care to reproduction) or 2–110 (reproduction to brood care). The optimal cluster size was determined to be the cluster with the lowest BIC score after stabilization to the plateau seen on the right of each graph. Arrows show the cluster selected for each transition. (PDF 53 kb)

Additional file 5: Summary of clusters enriched for DEGs. (PDF 29 kb)

Additional file 6: Basic network statistics of gene clusters for time course gene expression between both reproductive and brood care phase transitions. (PDF 51 kb)

Additional file 7: GO terms significantly enriched in clusters enriched for DEGs. Only two of these GO terms were common to both transitions (RB: transition from reproduction to brood care; BR: transition from brood care to reproduction). The diameter of the circles is proportional to the number of enriched GO terms. (PDF 97 kb)

Additional file 8: The conserved network (with only non-random connections) shows a lower proportion of edges linking clusters of genes regulated in opposite direction compared to the complete network (which includes random connections) (*χ*^2^ = 22.4, *p* < 0.00001). This finding is inconsistent with the same genes regulating both transitions. (PDF 47 kb)

Additional file 9: Top 40 DEGs (ranked according to log2 fold change in expression for control vs 12-h time point and control vs 96-h time point for each transition). (PDF 50 kb)

Additional file 10: Summary of clusters enriched for differentially expressed genes (DEGs) and transcription factor binding sites. (PDF 53 kb)

Additional file 11: Genes associated with *forkhead* also segregate with position in the colony cycle. Heatmap showing Euclidean distance between all samples for the 438 genes that contained at least one transcription factor binding site for *forkhead* with a minimum score of 95%. The dendrogram was constructed using the average distances between samples. The blue and green color bar above the heatmap indicates average ovary activation score, as in Fig. 2a. Sample names are as per Fig. 1. (PDF 49 kb)

Additional file 12: Number of replicates in the analyses (after outlier removal). (PDF 28 kb)

Additional file 13: Ovary activation scores. (XLSX 11 kb)
