# Supplementary figures and images for: Clonal raider ant brain transcriptomics identifies candidate molecular mechanisms for reproductive division of labor
Source: BMC Biol. 2018 Aug 13;16:89. doi: 10.1186/s12915-018-0558-8 (PMC6090591; doi:10.1186/s12915-018-0558-8)

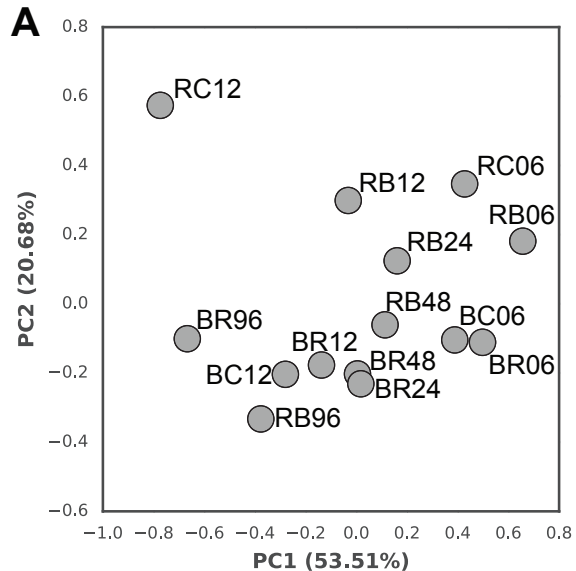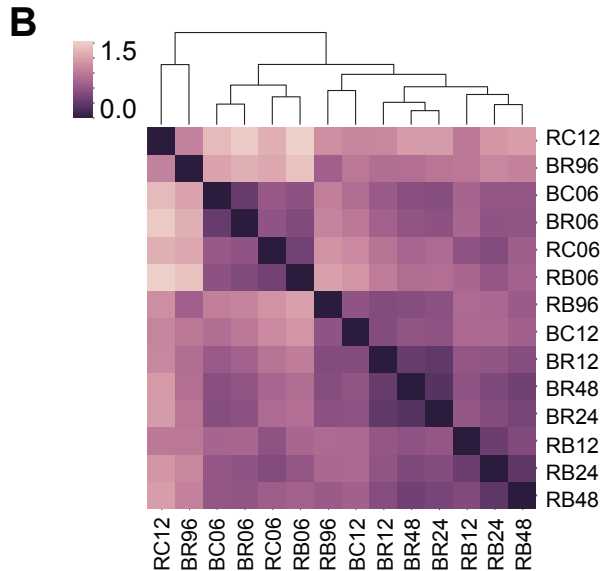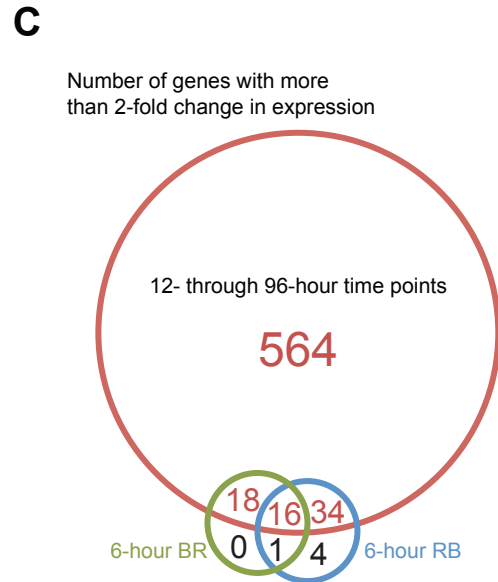

Supplement: Supplementary file 1 — Outlier analysis. PCA and distance map of genes showing greater than twofold change in expression. A) PCA plot of brood-swap and control samples. Clustering was based on the mean gene expression of each group, for 967 genes with more than twofold change in expression between samples. Percentages on each axis indicate the proportion of variance explained by the indicated principal component. The color of each sample indicates the expected similarity to the control samples; dark blue indicates reproductive phase and dark green indicates brood care phase. Sample names are as per Fig. 1. B) Heatmap showing Euclidean distance between all samples, based on all genes with more than twofold change in expression, and clustered according to average distances between samples. Blue and green color bar above the heatmap indicates similarity to control samples, as in A. Sample names are as per Fig. 1. C) Venn diagram showing outcome of eliminating the 6-h time points. Numbers in the small circles indicate genes with greater than twofold change in expression between 6-h control and treatment samples in the reproduction to brood care transition (blue) and brood care to reproduction transition (green) (a priori true positives). Red numbers indicate genes that show greater than twofold change in expression after removal of the 6-h time points. Thus, elimination of the 6-h samples does not substantially reduce the number of DEGs identified with large expression changes. (PDF 79 kb) [file 12915_2018_558_MOESM1_ESM.pdf]

### Brood care to reproduction

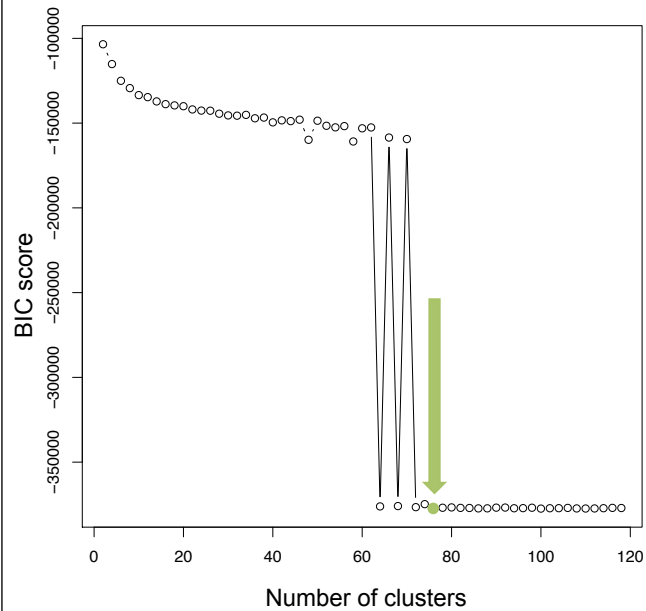

### Reproduction to brood care

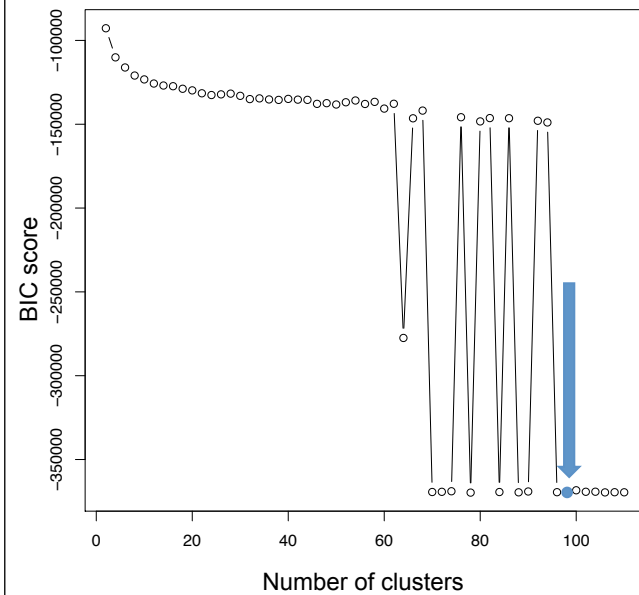

Supplement: Supplementary file 4 — Evaluation of BIC scores for selection of optimal number of clusters. Genes were clustered into all even numbered cluster sizes between 2 and 120 (brood care to reproduction) or 2–110 (reproduction to brood care). The optimal cluster size was determined to be the cluster with the lowest BIC score after stabilization to the plateau seen on the right of each graph. Arrows show the cluster selected for each transition. (PDF 53 kb) [file 12915_2018_558_MOESM4_ESM.pdf]

Proportion of edges linking clusters between transitions

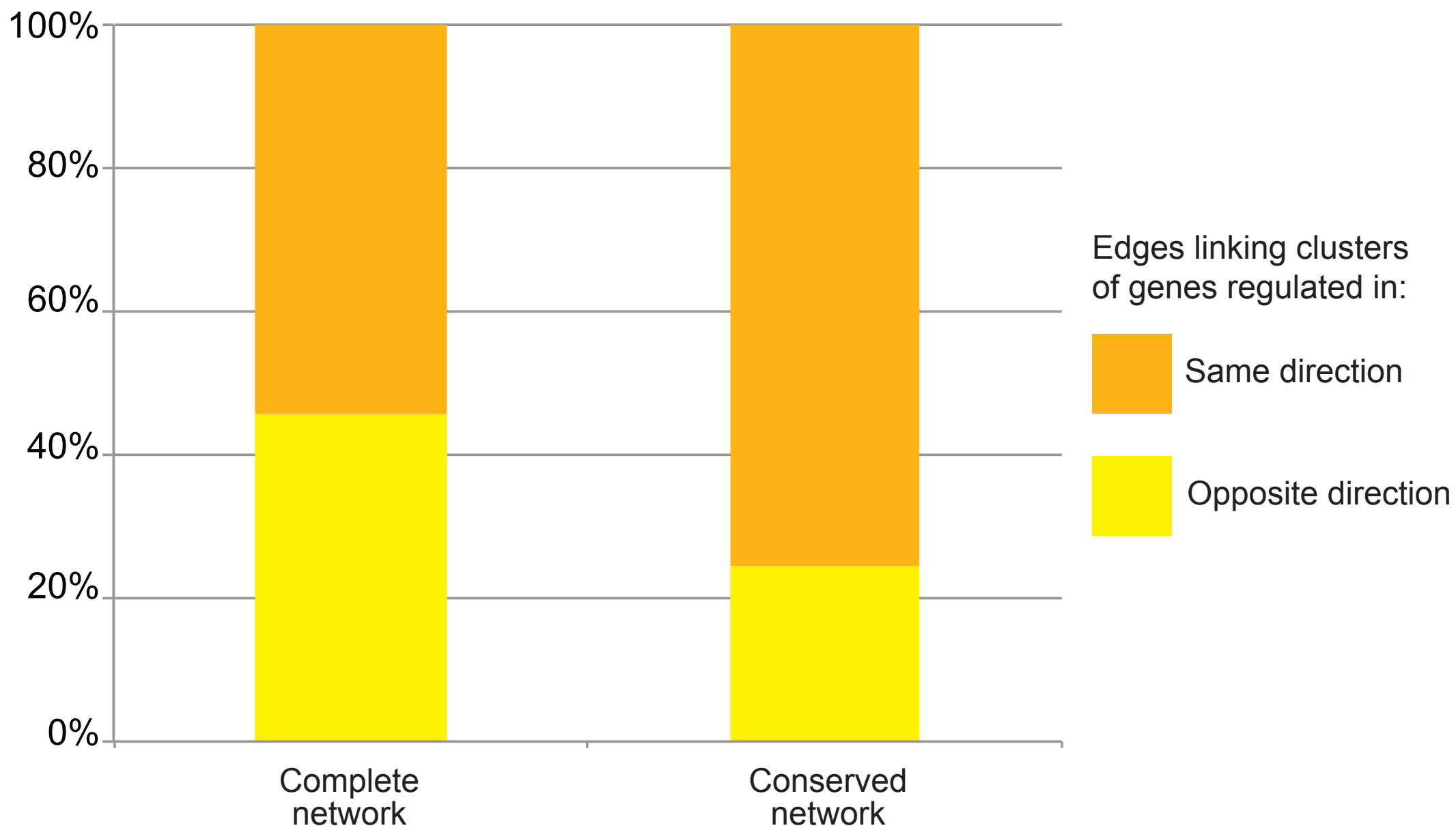

Supplement: Supplementary file 8 — The conserved network (with only non-random connections) shows a lower proportion of edges linking clusters of genes regulated in opposite direction compared to the complete network (which includes random connections) (χ2 = 22.4, p < 0.00001). This finding is inconsistent with the same genes regulating both transitions. (PDF 47 kb) [file 12915_2018_558_MOESM8_ESM.pdf]

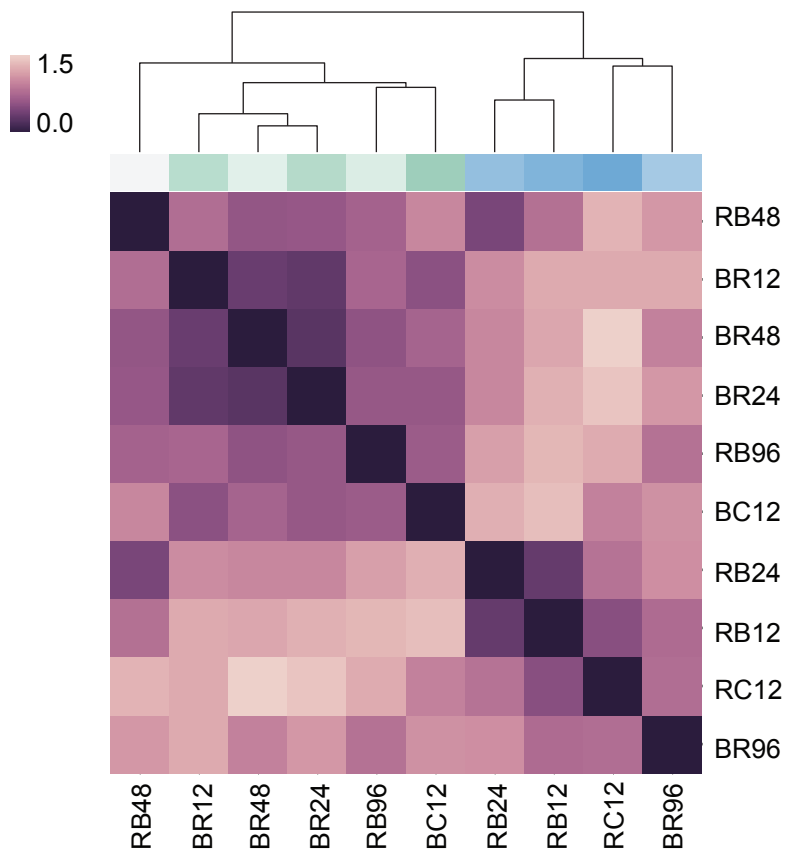

Supplement: Supplementary file 11 — Genes associated with forkhead also segregate with position in the colony cycle. Heatmap showing Euclidean distance between all samples for the 438 genes that contained at least one transcription factor binding site for forkhead with a minimum score of 95%. The dendrogram was constructed using the average distances between samples. The blue and green color bar above the heatmap indicates average ovary activation score, as in Fig. 2a. Sample names are as per Fig. 1. (PDF 49 kb) [file 12915_2018_558_MOESM11_ESM.pdf]
