# Supplementary material for: Clonal raider ant brain transcriptomics identifies candidate molecular mechanisms for reproductive division of labor
Source: BMC Biol. 2018 Aug 13;16:89. doi: 10.1186/s12915-018-0558-8 (PMC6090591; doi:10.1186/s12915-018-0558-8)
Supplement: Supplementary file 2 — All 596 DEGs ranked according to p value (smaller to larger). (PDF 101 kb) [file 12915_2018_558_MOESM2_ESM.pdf]

Rank	Gene ID	Adjusted p-value	Annotation
1	LOC105277456	2.86E-27	protein-L-isoaspartate(D-aspartate) O-methyltransferase
2	LOC105277457	2.09E-24	equilibrative nucleoside transporter 1
3	LOC105278524	5.91E-18	ILP2
4	LOC105281428_Q	1.25E-16	queen vitellogenin
5	LOC105275236	1.75E-16	carboxypeptidase M
6	LOC105284539	2.37E-12	sodium- and chloride-dependent glycine transporter 1
7	LOC105279766	2.72E-12	uncharacterized LOC105279766
8	LOC105279851	2.05E-11	yrdC domain-containing protein
9	LOC105275986	8.19E-11	uncharacterized LOC105275985
10	LOC105276495	1.17E-10	general odorant-binding protein 69a
11	LOC105285597	3.44E-10	uncharacterized LOC105285597
12	LOC105281011	1.12E-09	transferrin
13	LOC105285466	2.11E-09	uncharacterized LOC105285466
14	LOC105283669	7.35E-09	spermine synthase
15	LOC105276905	8.06E-09	neural/ectodermal development factor IMP-L2
16	LOC105275762	1.75E-08	protein aubergine
17	LOC105280170	4.95E-08	PI-PLC X domain-containing protein 2
18	LOC105281012	4.95E-08	uncharacterized LOC105281012
19	LOC105285380	6.73E-08	chitooligosaccharidolytic beta-N-acetylglucosaminidase
20	LOC105277400	2.52E-07	aldose 1-epimerase
21	LOC105279907	4.37E-07	uncharacterized LOC105279907
22	LOC105285376	4.71E-07	uncharacterized LOC105285376
23	LOC105280731	5.52E-07	inositol monophosphatase 1
24	LOC105275113	8.88E-07	pancreatic lipase-related protein 2-like
25	LOC105276066	1.03E-06	uncharacterized LOC105276066
26	LOC105275269	1.09E-06	uncharacterized LOC105275269
27	LOC105276731	1.09E-06	uncharacterized LOC105276731
28	LOC105283821	1.09E-06	nucleolar protein 6
29	LOC105278211	1.54E-06	centromere protein I
30	LOC105276018	1.94E-06	peroxisomal hydratase-dehydrogenase-epimerase
31	LOC105277056	1.94E-06	neuroparsin-A
32	LOC105288064	3.80E-06	uncharacterized LOC105288064
33	LOC105281928	5.80E-06	L-asparaginase 1
34	LOC105285607	6.17E-06	probable ribosome production factor 1
35	LOC105286129	6.88E-06	homeobox protein TGIF2LX
36	LOC105280465	9.64E-06	uncharacterized LOC105280465
37	LOC105281340	9.64E-06	sphingomyelin phosphodiesterase
38	LOC105283223	9.64E-06	putative mediator of RNA polymerase II transcription subunit 26
39	LOC105279511	9.67E-06	uncharacterized LOC105279511
40	LOC105281817	1.08E-05	uncharacterized LOC105281817
41	LOC105286755	1.08E-05	peptidyl-prolyl cis-trans isomerase 5
42	LOC105282988	1.18E-05	sodium/potassium-transporting ATPase subunit alpha
43	LOC105277203	1.24E-05	RNA polymerase I-specific transcription initiation factor RRN3
44	LOC105281008	1.45E-05	glutamate receptor ionotropic
45	LOC105286732	1.65E-05	anillin
46	LOC105281609	1.97E-05	small subunit processome component 20 homolog
47	LOC105285940	2.17E-05	THAP domain-containing protein 3
48	LOC105281708	2.20E-05	uncharacterized LOC105281708
49	LOC105281726	2.43E-05	ferrochelatase
50	LOC105280087	2.72E-05	protein regulator of cytokinesis 1
51	LOC105279867	2.74E-05	growth factor receptor-bound protein 14
52	LOC105283177	4.99E-05	glutathione S-transferase
53	LOC105276918	5.07E-05	14-3-3 protein epsilon
54	LOC105285920	5.07E-05	vegetative cell wall protein gp1
55	LOC105276675	5.36E-05	NK-tumor recognition protein
56	LOC105284285	5.36E-05	high affinity cAMP-specific and IBMX-insensitive 3'
57	LOC105280553	5.43E-05	uncharacterized LOC105280553
58	LOC105279540	5.72E-05	N-sulphoglucosamine sulphohydrolase
59	LOC105282075	5.77E-05	structural maintenance of chromosomes protein 5
60	LOC105282643	7.25E-05	PDZ and LIM domain protein 4
61	LOC105283185	7.50E-05	kinetochore-associated protein 1
62	LOC105287151	8.77E-05	D-beta-hydroxybutyrate dehydrogenase
63	LOC105286142	9.53E-05	cyclin-dependent kinases regulatory subunit
64	LOC105277367	0.000105047	twinkle protein
65	LOC105283914	0.000105319	mitochondrial import receptor subunit TOM70

66	LOC105282416	0.000109464	uncharacterized LOC105282416
67	LOC105277606	0.000118589	titin
68	LOC105285641	0.000129828	EP300-interacting inhibitor of differentiation 3
69	LOC105286010	0.000132779	major facilitator superfamily domain-containing protein 12
70	LOC105287874	0.000145545	NADH-ubiquinone oxidoreductase 49 kDa subunit
71	LOC105276419	0.000151946	uncharacterized protein C18orf63
72	LOC105282036	0.000151946	G2/mitotic-specific cyclin-B
73	LOC105282150	0.000151946	ATP synthase subunit alpha
74	LOC105275344	0.000168939	ejaculatory bulb-specific protein 3
75	LOC105276174	0.000175408	sodium/potassium-transporting ATPase subunit beta-2
76	LOC105278710	0.000176586	cyclin-dependent kinase 1
77	LOC105281832	0.000181294	nucleolar protein 58
78	LOC105279728	0.000206086	uncharacterized LOC105279728
79	LOC105279314	0.000213503	trypsin inhibitor
80	LOC105287013	0.000213503	kinesin-like protein KIF20B
81	LOC105280074	0.000220032	uncharacterized LOC105280074
82	LOC105275711	0.000229201	TATA box-binding protein-associated factor RNA polymerase I subunit B
83	LOC105280641	0.000231988	serine/threonine-protein kinase VRK1
84	LOC105279847	0.000235166	uncharacterized LOC105279847
85	LOC105282680	0.00025994	glucosylceramidase
86	LOC105279763	0.000261952	glutamate receptor ionotropic
87	LOC105279565	0.000268443	transcriptional regulator ATRX
88	LOC105288139	0.000292672	uncharacterized LOC105288139
89	LOC105279557	0.000301454	kinesin-like protein KIF19
90	LOC105283374	0.000301454	poly(A) RNA polymerase gld-2 homolog A
91	LOC105286171	0.000319218	contactin-4
92	LOC105285834	0.000381783	ATP-dependent DNA helicase Q5
93	LOC105274638	0.000388134	kinesin-like protein KIF18A
94	LOC105284189	0.000389058	fidgetin-like protein 1
95	LOC105285614	0.000413081	glycerol-3-phosphate dehydrogenase [NAD(+)]
96	LOC105274740	0.000434313	putative leucine-rich repeat-containing protein DDB_G0290503
97	LOC105277839	0.000434313	ribosomal protein S6 kinase beta-1
98	LOC105281660	0.000442336	interleukin enhancer-binding factor 2
99	LOC105276143	0.000465598	NGFI-A-binding protein homolog
100	LOC105277886	0.000499745	glutamate-gated chloride channel
101	LOC105279186	0.000549461	probable RNA-binding protein 19
102	LOC105280308	0.000549461	Fanconi anemia group D2 protein homolog
103	LOC105280529	0.000549461	uncharacterized LOC105280529
104	LOC105282076	0.000549461	small lysine-rich protein 1
105	LOC105286277	0.000549461	palmitoyltransferase ZDHHC3
106	LOC105275889	0.000572928	uncharacterized LOC105275889
107	LOC105275854	0.000582604	annulin
108	LOC105276290	0.000582604	nuclear pore complex protein Nup88
109	LOC105280511	0.000585378	glucose dehydrogenase [FAD
110	LOC105286952	0.000608702	uncharacterized LOC105286952
111	LOC105285125	0.000611241	endoribonuclease Dicer
112	LOC105282639	0.000620718	lachesin
113	LOC105275876	0.000649572	diuretic hormone class 2
114	LOC105279470	0.000649572	signal peptidase complex subunit 1
115	LOC105277844	0.000663379	cytochrome P450 CYP12A2
116	LOC105281212	0.000702038	protein rhomboid
117	LOC105283509	0.000717333	zinc finger BED domain-containing protein 1-like
118	LOC105276079	0.000720172	uncharacterized LOC105276079
119	LOC105286126	0.000720172	succinate dehydrogenase [ubiquinone] iron-sulfur subunit
120	LOC105283079	0.000755324	midasin
121	LOC105276628	0.000766774	uncharacterized MFS-type transporter C09D4.1
122	LOC105278567	0.000766774	zinc finger BED domain-containing protein 1
123	LOC105283043	0.000775008	uncharacterized LOC105283043
124	LOC105283843	0.000775008	phosphatidylinositol transfer protein alpha isoform
125	LOC105286179	0.000775008	uncharacterized LOC105286179
126	LOC105288016	0.000775008	synaptic vesicular amine transporter
127	LOC105276979	0.000791533	isocitrate dehydrogenase [NAD] subunit beta
128	LOC105286439	0.000826073	uncharacterized LOC105286439
129	LOC105277700	0.000859212	actin
130	LOC105274628	0.000891361	uncharacterized LOC105274628
131	LOC105280285	0.00089511	uncharacterized protein CG1785
132	LOC105288014	0.000939241	HEAT repeat-containing protein 1

133	LOC105281721	0.000973321	uncharacterized LOC105281721
134	LOC105283157	0.000973321	carbonic anhydrase 2
135	LOC105283626	0.000973321	uncharacterized LOC105283626
136	LOC105287187	0.000973321	non-homologous end-joining factor 1
137	LOC105276512	0.00103448	S-methyl-5'-thioadenosine phosphorylase
138	LOC105277421	0.00103448	protein timeless homolog
139	LOC105279939	0.001075557	lymphokine-activated killer T-cell-originated protein kinase
140	LOC105276690	0.001096309	folylpolyglutamate synthase
141	LOC105275469	0.001107455	sarcalumenin
142	LOC105285890	0.001107455	uncharacterized LOC105285890
143	LOC105283266	0.001121027	lymphoid-specific helicase
144	LOC105276925	0.001123963	protein seele
145	LOC105287204	0.001123963	twitchin
146	LOC105276190	0.001155579	protein brambleberry
147	LOC105279559	0.001155579	glycine receptor subunit alpha-2-like
148	LOC105280561	0.001172392	calmodulin
149	LOC105278661	0.001211963	ribonucleases P/MRP protein subunit POP1
150	LOC105278082	0.001262696	ADP-ribosylation factor-like protein 13B
151	LOC105284604	0.001354385	general odorant-binding protein 83a
152	LOC105287093	0.001396307	uncharacterized LOC105287093
153	LOC105284249	0.001459572	ribosomal RNA processing protein 1 homolog
154	LOC105284986	0.001480837	ribonucleoside-diphosphate reductase large subunit
155	LOC105283698	0.001521001	pyridoxine-5'-phosphate oxidase
156	LOC105282844	0.001531753	uncharacterized LOC105282844
157	LOC105276853	0.001584309	uncharacterized LOC105276853
158	LOC105287282	0.001590122	nucleotide exchange factor SIL1
159	LOC105285386	0.00171934	nucleoporin Nup37
160	LOC105275345	0.001775821	ejaculatory bulb-specific protein 3
161	LOC105287530	0.001775821	WD repeat-containing protein 46
162	LOC105279696	0.001802057	uncharacterized LOC105279696
163	LOC105280503	0.001861233	glucose dehydrogenase [FAD
164	LOC105286339	0.001892283	RRP12-like protein
165	LOC105277377	0.001910982	troponin C
166	LOC105279473	0.001962256	uncharacterized LOC105279473
167	LOC105280338	0.001962256	CAD protein
168	LOC105277875	0.00197776	RNA-binding protein 40
169	LOC105281005	0.001994437	tubulin epsilon chain
170	LOC105287193	0.002024743	uncharacterized LOC105287193
171	LOC105285809	0.002063242	putative helicase MOV-10
172	LOC105285704	0.002174642	MATH and LRR domain-containing protein PFE0570w
173	LOC105279590	0.002190403	uncharacterized LOC105279590
174	LOC105279671	0.002197433	U3 small nucleolar RNA-associated protein 14 homolog A
175	LOC105285187	0.002212392	uncharacterized LOC105285187
176	LOC105277039	0.002257698	CAPA peptides
177	LOC105277876	0.002257698	adenylate kinase isoenzyme 1
178	LOC105280242	0.002257698	uncharacterized LOC105280240
179	LOC105283045	0.002257698	uncharacterized LOC105283045
180	LOC105283429	0.002257698	uridine phosphorylase 1
181	LOC105286263	0.002257698	ribonuclease P protein subunit p40
182	LOC105278693	0.002374317	troponin C-like
183	LOC105286776	0.002374317	ribonucleoside-diphosphate reductase subunit M2 B
184	LOC105283233	0.002468175	fibrillin-1
185	LOC105284536	0.002470555	uncharacterized LOC105284536
186	LOC105287615	0.002622097	nidogen-2
187	LOC105276738	0.002627827	KH domain-containing protein akap-1
188	LOC105284373	0.002714911	myosin light chain alkali
189	LOC105287207	0.002888659	kinesin-like protein Klp61F
190	LOC105285677	0.003023458	uncharacterized LOC105285677
191	LOC105285139	0.003043257	serine/threonine-protein kinase Chk2
192	LOC105277197	0.00317964	putative sodium-dependent multivitamin transporter
193	LOC105277432	0.003195212	V-type proton ATPase subunit G
194	LOC105287079	0.003237844	protein CNPPD1
195	LOC105280404	0.003258013	tankyrase-1
196	LOC105286412	0.003258013	uncharacterized LOC105286412
197	LOC105283216	0.003309992	mycosubtilin synthase subunit C
198	LOC105274480	0.003328645	zwei Ig domain protein zig-8
199	LOC105280897	0.003328645	titin

200	LOC105278020	0.003375831	origin recognition complex subunit 2
201	LOC105283438	0.003471681	tyrosine--tRNA ligase
202	LOC105278746	0.003508497	periodic tryptophan protein 1 homolog
203	LOC105284104	0.003563795	uncharacterized LOC105284104
204	LOC105285852	0.003563795	nucleoside diphosphate kinase 6
205	LOC105283164	0.003564436	protein FAM60A
206	LOC105284641	0.003564436	uncharacterized LOC105284641
207	LOC105286766	0.003564436	dynein heavy chain 7
208	LOC105282386	0.003627323	tRNA-specific adenosine deaminase 2
209	LOC105281770	0.003726834	phosphoglycerate mutase 1
210	LOC105286356	0.003733126	R3H and coiled-coil domain-containing protein 1
211	LOC105285273	0.003745615	UDP-glucuronosyltransferase 2B1
212	LOC105276762	0.003752298	chaoptin
213	LOC105286471	0.003786951	uncharacterized LOC105286471
214	LOC105286745	0.003786951	serine proteinase stubble
215	LOC105287773	0.003786951	uncharacterized LOC105287773
216	LOC105281565	0.0039943	maternal embryonic leucine zipper kinase
217	LOC105281396	0.00400507	angiotensin-converting enzyme 2
218	LOC105278260	0.004033359	tRNA-splicing ligase RtcB homolog
219	LOC105282051	0.004069005	uncharacterized LOC105282051
220	LOC105284988	0.004069005	DNA primase large subunit
221	LOC105277716	0.004092585	facilitated trehalose transporter Tret1
222	LOC105278265	0.004107218	uncharacterized LOC105278265
223	LOC105282754	0.004152694	protein yellow
224	LOC105284776	0.004152694	uncharacterized LOC105284776
225	LOC105275039	0.004204795	uncharacterized LOC105275039
226	LOC105275568	0.004204795	probable pyruvate dehydrogenase E1 component subunit alpha
227	LOC105285003	0.004266796	uncharacterized LOC105285003
228	LOC105276507	0.004334577	notchless protein homolog 1
229	LOC105275184	0.004373376	SWI/SNF-related matrix-associated actin-dependent regulator of chromatin subfamily A-like protein 1
230	LOC105276445	0.004373376	la protein homolog
231	LOC105279818	0.004373376	G2/mitotic-specific cyclin-B3
232	LOC105281245	0.004373376	uncharacterized LOC105281245
233	LOC105285918	0.004373376	probable kinetochore protein NUF2
234	LOC105286949	0.004373376	RNA-binding protein 4.1
235	LOC105286220	0.004374269	nuclear pore complex protein Nup133
236	LOC105278869	0.00442564	uncharacterized LOC105278869
237	LOC105277651	0.004438611	rap1 GTPase-activating protein 1
238	LOC105286343	0.004658075	putative serine protease K12H4.7
239	LOC105283179	0.004746696	carcinine transporter
240	LOC105281685	0.004888436	venom carboxylesterase-6
241	LOC105280491	0.004955404	inosine-5'-monophosphate dehydrogenase
242	LOC105277108	0.004990146	uncharacterized LOC105277108
243	LOC105277299	0.004990146	ionotropic receptor 25a
244	LOC105278142	0.00501094	protein phosphatase methylesterase 1
245	LOC105282563	0.005136377	general transcription factor IIH subunit 1
246	LOC105283753	0.005136377	matrix metalloproteinase-2
247	LOC105287183	0.005136377	uncharacterized LOC105287183
248	LOC105286143	0.005213679	renin receptor
249	LOC105275836	0.005222883	dexamethasone-induced Ras-related protein 1
250	LOC105279586	0.005250814	uncharacterized LOC105279586
251	LOC105284384	0.005250814	protein stum
252	LOC105279197	0.005273064	cyclin-dependent-like kinase 5
253	LOC105277469	0.005407787	tubulin beta chain
254	LOC105285464	0.0055311	calmodulin-binding transcription activator 2
255	LOC105280121	0.00553732	esterase E4
256	LOC105277435	0.005768748	targeting protein for Xklp2
257	LOC105277439	0.005787527	ADP-ribosylation factor-like protein 6
258	LOC105279165	0.005861519	LIM domain and actin-binding protein 1
259	LOC105287714	0.005943601	protein hunchback
260	LOC105274872	0.006089499	V-type proton ATPase subunit B
261	LOC105275324	0.006089499	angio-associated migratory cell protein
262	LOC105286103	0.006237383	glutamate--cysteine ligase catalytic subunit
263	LOC105284633	0.006238679	gliomedin
264	LOC105279018	0.006440394	ras-related and estrogen-regulated growth inhibitor
265	LOC105276398	0.006458399	beta-hexosaminidase subunit beta
266	LOC105276421	0.006458399	reticulon-4-interacting protein 1

267	LOC105277488	0.006458399	neuropeptide CCHamide-2 receptor-like
268	LOC105282470	0.006458399	centromere protein L
269	LOC105284938	0.006505284	telomerase reverse transcriptase-like
270	LOC105281051	0.006549854	protein FAM173B
271	LOC105279471	0.00663211	uncharacterized LOC105279471
272	LOC105281618	0.006925192	uncharacterized LOC105281618
273	LOC105284524	0.0069971	hemocytin
274	LOC105280836	0.00727081	uncharacterized LOC105280836
275	LOC105274893	0.007357978	uncharacterized LOC105274893
276	LOC105286889	0.007357978	uncharacterized LOC105286889
277	LOC105274660	0.007463435	uncharacterized LOC105274660
278	LOC105274901	0.007463435	SH3 domain-binding glutamic acid-rich protein homolog
279	LOC105280103	0.007479243	inositol-tetrakisphosphate 1-kinase
280	LOC105277350	0.007511244	uncharacterized LOC105277350
281	LOC105284074	0.007705767	phospholipase B1
282	LOC105285671	0.007705767	nucleoporin Ndc1
283	LOC105283621	0.007727391	synaptic vesicle glycoprotein 2C
284	LOC105280985	0.007749383	ATP-dependent RNA helicase SUV3 homolog
285	LOC105276851	0.007925953	rho guanine nucleotide exchange factor 10-like protein
286	LOC105277458	0.007925953	DNA replication licensing factor MCM4
287	LOC105281382	0.007996421	deoxynucleoside kinase
288	LOC105279911	0.00811899	sterol O-acyltransferase 1
289	LOC105287024	0.008305523	uncharacterized LOC105287024
290	LOC105275955	0.008312762	DNA topoisomerase 2-binding protein 1-A
291	LOC105278498	0.008312762	neither inactivation nor afterpotential protein G
292	LOC105278931	0.008312762	venom allergen 3
293	LOC105279816	0.008312762	cAMP-specific 3'
294	LOC105285550	0.008312762	heat shock 70 kDa protein cognate 4
295	LOC105287679	0.008312762	kinetochore protein NDC80 homolog
296	LOC105276789	0.008448158	putative methyltransferase NSUN6
297	LOC105286365	0.008459175	uncharacterized LOC105286365
298	LOC105278774	0.008516871	hydroxypyruvate reductase
299	LOC105279012	0.008712235	uncharacterized LOC105279012
300	LOC105283008	0.008867065	uncharacterized LOC105283008
301	LOC105282359	0.008886148	myosin regulatory light chain 2
302	LOC105276807	0.009003188	mitochondrial-processing peptidase subunit beta
303	LOC105286101	0.009100546	uncharacterized LOC105286101
304	LOC105286139	0.009113335	translation elongation factor 2
305	LOC105282872	0.009170765	acylphosphatase-1
306	LOC105285256	0.009279029	copper chaperone for superoxide dismutase
307	LOC105288172	0.009328315	hepatocyte nuclear factor 6-like
308	LOC105281757	0.00959872	type I inositol 1
309	LOC105282576	0.01011098	U3 small nucleolar RNA-interacting protein 2
310	LOC105285936	0.010130592	glutathione S-transferase 1-1
311	LOC105281321	0.010183213	5-phosphohydroxy-L-lysine phospho-lyase
312	LOC105284488	0.010203996	meiosis arrest female protein 1 homolog
313	LOC105277205	0.010456356	aquaporin-12A
314	LOC105278655	0.010905246	ATP synthase lipid-binding protein
315	LOC105284448	0.010964398	uncharacterized LOC105284448
316	LOC105281030	0.010989901	uncharacterized LOC105281030
317	LOC105284205	0.011084639	nucleolar GTP-binding protein 1
318	LOC105280772	0.011298076	uncharacterized LOC105280772
319	LOC105282573	0.011350785	SH2B adapter protein 1
320	LOC105279271	0.011467688	uncharacterized LOC105279271
321	LOC105283206	0.011714415	THAP domain-containing protein 5-like
322	LOC105276279	0.011796248	dynein regulatory complex protein 1
323	LOC105283005	0.012137093	protein lethal(2)essential for life
324	LOC105277437	0.013010433	protein LTV1 homolog
325	LOC105283737	0.013047134	myosin heavy chain
326	LOC105288114	0.013047134	replication factor C subunit 4
327	LOC105277654	0.013069808	uncharacterized LOC105277654
328	LOC105275475	0.013180822	bifunctional methylenetetrahydrofolate dehydrogenase/cyclohydrolase
329	LOC105283200	0.013180822	embryonic polarity protein dorsal
330	LOC105275339	0.01329855	H/ACA ribonucleoprotein complex subunit 4
331	LOC105277860	0.013408944	monocarboxylate transporter 12
332	LOC105277685	0.013431626	solute carrier family 12 member 4
333	LOC105286216	0.01354877	kinesin-like protein KIF23

334	LOC105284533	0.013556775	RISC-loading complex subunit tarbp2
335	LOC105276678	0.013571358	transferrin
336	LOC105281010	0.013571358	uncharacterized LOC105281010
337	LOC105282771	0.013571358	exosome complex component RRP40
338	LOC105276745	0.013910074	pre-rRNA processing protein FTSJ3
339	LOC105281448	0.013910074	twitchin
340	LOC105274926	0.014081898	RNA-binding protein 34
341	LOC105277818	0.014081898	uncharacterized LOC105277818
342	LOC105275821	0.014102391	dnaJ homolog subfamily C member 22
343	LOC105286289	0.014120423	upstream stimulatory factor 2
344	LOC105282390	0.01455199	single-strand selective monofunctional uracil DNA glycosylase
345	LOC105288106	0.01455199	sarcosine dehydrogenase
346	LOC105281668	0.014858442	E3 ubiquitin-protein ligase RFWD2-like
347	LOC105277939	0.015013487	uncharacterized LOC105277939
348	LOC105283516	0.015070799	dual oxidase 2
349	LOC105283205	0.015279418	ATP-dependent RNA helicase Ddx1
350	LOC105282422	0.016180145	cuticle protein 64
351	LOC105274940	0.016450608	homeobox protein prospero
352	LOC105279513	0.016450608	tRNA dimethylallyltransferase
353	LOC105280398	0.016525208	protein will die slowly
354	LOC105279732	0.016535081	chymotrypsin-like elastase family member 2A
355	LOC105287010	0.016601834	uncharacterized LOC105287010
356	LOC105283803	0.016726746	ATP synthase subunit O
357	LOC105278140	0.016770953	BTB/POZ domain-containing protein KCTD16
358	LOC105280581	0.016868053	coiled-coil domain-containing protein 50
359	LOC105281377	0.016956368	uncharacterized LOC105281377
360	LOC105278656	0.017140571	solute carrier family 25 member 40
361	LOC105278757	0.017140571	uncharacterized LOC105278757
362	LOC105287066	0.017519339	DNA ligase 1
363	LOC105287949	0.017530134	protein LLP homolog
364	LOC105283796	0.017845437	ornithine decarboxylase 2-like
365	LOC105278078	0.017864174	uncharacterized LOC105278078
366	LOC105287636	0.017896942	uncharacterized LOC105287636
367	LOC105278194	0.0180767	prolyl 3-hydroxylase OGFOD1
368	LOC105285921	0.018086746	uncharacterized LOC105285921
369	LOC105282866	0.018105674	myb-binding protein 1A
370	LOC105275582	0.018212091	zinc finger protein 275
371	LOC105280537	0.018304925	uncharacterized LOC105280537
372	LOC105275811	0.01861386	ribosome biogenesis protein BOP1 homolog
373	LOC105280125	0.01861386	uncharacterized LOC105280125
374	LOC105281082	0.01861386	uncharacterized LOC105281082
375	LOC105286381	0.018645702	uncharacterized LOC105286381
376	LOC105277441	0.018874679	mucin-2
377	LOC105283628	0.018918359	uncharacterized LOC105283628
378	LOC105280004	0.019061425	dynein assembly factor 3
379	LOC105284779	0.019110966	uncharacterized LOC105284779
380	LOC105280988	0.019550262	ejaculatory bulb-specific protein 3
381	LOC105284119	0.019585838	ribonuclease H-like
382	LOC105280397	0.019846382	DNA-dependent protein kinase catalytic subunit
383	LOC105282816	0.020174174	uncharacterized LOC105282816
384	LOC105276699	0.020382878	uncharacterized LOC105276699
385	LOC105285953	0.020382878	uncharacterized LOC105285953
386	LOC105275455	0.020403346	ankyrin repeat and LEM domain-containing protein 2
387	LOC105277915	0.020403346	probable ATP-dependent RNA helicase DDX17
388	LOC105280774	0.020403346	rho guanine nucleotide exchange factor 3
389	LOC105283366	0.020403346	uncharacterized LOC105283366
390	LOC105285712	0.020403346	tolloid-like protein 2
391	LOC105286720	0.020403346	nucleolar protein 56
392	LOC105282918	0.020409442	putative fatty acyl-CoA reductase CG5065
393	LOC105287587	0.020806822	uncharacterized LOC105287587
394	LOC105274629	0.020916032	uncharacterized LOC105274629
395	LOC105282320	0.020916032	hemicentin-2
396	LOC105276546	0.020954818	uncharacterized LOC105276546
397	LOC105275970	0.02108041	uncharacterized LOC105275970
398	LOC105284165	0.021328675	protein THEM6
399	LOC105285353	0.021510104	uncharacterized LOC105285353
400	LOC105281103	0.021841118	pancreatic triacylglycerol lipase-like

401	LOC105277131	0.022127761	uncharacterized LOC105277131
402	LOC105287812	0.022255454	uncharacterized LOC105287812
403	LOC105284497	0.02226819	intraflagellar transport protein 46 homolog
404	LOC105285007	0.022298915	uncharacterized LOC105285007
405	LOC105277337	0.022352653	tubulin polyglutamylase TTLL4
406	LOC105283527	0.023808013	uncharacterized LOC105283527
407	LOC105285553	0.023808013	uncharacterized LOC105285553
408	LOC105287684	0.023842556	uncharacterized LOC105287684
409	LOC105287283	0.023980629	nucleoplasmin-like protein
410	LOC105277137	0.024077995	protein lethal(2)essential for life
411	LOC105283368	0.024691043	probable ATP-dependent RNA helicase CG8611
412	LOC105284077	0.024988634	juvenile hormone epoxide hydrolase 1-like
413	LOC105279278	0.025129854	lysosomal thioesterase PPT2 homolog
414	LOC105282179	0.025129854	NADH dehydrogenase [ubiquinone] 1 beta subcomplex subunit 2
415	LOC105286407	0.025129854	titin
416	LOC105284466	0.025211033	NADH dehydrogenase [ubiquinone] 1 alpha subcomplex subunit 10
417	LOC105274997	0.025259326	rRNA methyltransferase 2
418	LOC105284252	0.025766983	uncharacterized LOC105284252
419	LOC105280202	0.026009546	glycine-rich cell wall structural protein
420	LOC105276085	0.026393569	uncharacterized LOC105276085
421	LOC105284185	0.026393569	probable RNA-binding protein EIF1AD
422	LOC105285503	0.026393569	protein tyrosine phosphatase domain-containing protein 1
423	LOC105281711	0.026450157	serine/threonine-protein phosphatase PP1-beta catalytic subunit
424	LOC105282531	0.026901222	mitochondrial pyruvate carrier 2
425	LOC105287273	0.026984143	E3 ubiquitin-protein ligase rnf8-A
426	LOC105276433	0.02717383	box A-binding factor
427	LOC105276873	0.02717383	protein tipE
428	LOC105281449	0.02717383	muscle M-line assembly protein unc-89-like
429	LOC105284115	0.027407734	uncharacterized LOC105284115
430	LOC105276534	0.027511855	uncharacterized LOC105276534
431	LOC105282149	0.027531068	guanine nucleotide-binding protein subunit beta-5
432	LOC105285652	0.027531068	proton-coupled amino acid transporter-like protein CG1139
433	LOC105287285	0.027531068	zinc transporter ZIP13 homolog
434	LOC105280369	0.02784133	uncharacterized LOC105280369
435	LOC105281610	0.027939891	origin recognition complex subunit 1
436	LOC105285829	0.028011009	guanine nucleotide-binding protein-like 3 homolog
437	LOC105286156	0.028011009	gamma-aminobutyric acid receptor subunit beta-like
438	LOC105285775	0.028055328	uncharacterized LOC105285775
439	LOC105282104	0.028088617	glutamate decarboxylase
440	LOC105277525	0.028172885	uncharacterized LOC105277525
441	LOC105286769	0.028172885	uncharacterized LOC105286769
442	LOC105278343	0.028273197	serine/threonine-protein phosphatase 6 regulatory ankyrin repeat subunit A
443	LOC105284789	0.028448938	uncharacterized LOC105284789
444	LOC105287552	0.028448938	protein snakeskin
445	LOC105275954	0.028521019	cytochrome P450 6B7-like
446	LOC105281037	0.029105349	microtubule-associated protein futsch
447	LOC105279447	0.029260078	poly(ADP-ribose) glycohydrolase ARH3
448	LOC105284794	0.029260078	uncharacterized LOC105284794
449	LOC105284925	0.029260078	chloride channel protein 2
450	LOC105276504	0.029409338	calcineurin subunit B type 2
451	LOC105278482	0.029581681	E3 ubiquitin-protein ligase TRAIP
452	LOC105278101	0.030503559	CCA tRNA nucleotidyltransferase 1
453	LOC105275507	0.03071899	PDZ and LIM domain protein 3
454	LOC105279588	0.030725831	activating signal cointegrator 1 complex subunit 3
455	LOC105286287	0.031079663	uncharacterized LOC105286287
456	LOC105288201	0.031079663	uncharacterized LOC105288201
457	LOC105284128	0.031224018	sialin
458	LOC105279871	0.031406262	oxysterol-binding protein-related protein 2
459	LOC105278676	0.031605854	uncharacterized LOC105278676
460	LOC105275286	0.031779213	innexin inx3
461	LOC105279699	0.03198123	prisilkin-39-like
462	LOC105278724	0.032342333	fatty acid synthase-like
463	LOC105278934	0.032342333	exonuclease 3'-5' domain-containing protein 2
464	LOC105285644	0.032342333	rootletin
465	LOC105287695	0.032342333	serine/threonine-protein kinase polo
466	LOC105278009	0.032494456	diuretic hormone 44
467	LOC105281176	0.032494456	uncharacterized LOC105281176

468	LOC105287539	0.032494456	protein vein
469	LOC105286140	0.032710688	zinc finger protein 474
470	LOC105288078	0.032728271	WD repeat-containing protein 43
471	LOC105278695	0.033077184	filamin-A
472	LOC105282856	0.033177527	N-acetyltransferase ESCO2
473	LOC105285465	0.033323481	AP-2 complex subunit mu
474	LOC105275769	0.033494647	uncharacterized LOC105275769
475	LOC105274965	0.0335862	cyclin-dependent kinase-like 4
476	LOC105279598	0.0335862	glutamate dehydrogenase
477	LOC105276193	0.034334734	uncharacterized LOC105276193
478	LOC105277743	0.034334734	trimethylguanosine synthase
479	LOC105287116	0.034334734	la-related protein 7
480	LOC105281786	0.034343523	hyaluronan mediated motility receptor
481	LOC105285243	0.034343523	lysozyme
482	LOC105277206	0.034521091	sodium- and chloride-dependent GABA transporter ine
483	LOC105285400	0.034714853	gem-associated protein 5
484	LOC105287758	0.034740117	uncharacterized LOC105287758
485	LOC105286522	0.034969332	alkylglycerol monooxygenase-like
486	LOC105279786	0.035783651	1-acyl-sn-glycerol-3-phosphate acyltransferase gamma
487	LOC105282695	0.035783651	surfeit locus protein 1
488	LOC105282710	0.035783651	protein penguin
489	LOC105283615	0.035783651	serine protease nudel
490	LOC105284985	0.035783651	putative uncharacterized protein DDB_G0271606
491	LOC105277279	0.035909715	probable citrate synthase 2
492	LOC105282173	0.036134281	homeotic protein ultrabithorax
493	LOC105283611	0.036243182	allatostatin A
494	LOC105277912	0.036320028	translation initiation factor eIF-2B subunit epsilon
495	LOC105281216	0.036320028	F-box only protein 21
496	LOC105285869	0.036539819	ubiquitin-conjugating enzyme E2 H
497	LOC105287550	0.036680683	serine/arginine repetitive matrix protein 1
498	LOC105280031	0.036693551	ankyrin repeat
499	LOC105280990	0.036693551	nanos homolog 3
500	LOC105281197	0.036849698	uncharacterized LOC105281197
501	LOC105277728	0.036980806	uncharacterized LOC105277728
502	LOC105279536	0.036980806	uncharacterized LOC105279536
503	LOC105286284	0.036980806	uncharacterized LOC105286284
504	LOC105274442	0.03720635	selenide
505	LOC105277683	0.03720635	bromodomain-containing protein 7
506	LOC105278758	0.03720635	actin
507	LOC105284134	0.03720635	uncharacterized LOC105284134
508	LOC105279916	0.037236442	major facilitator superfamily domain-containing protein 6-A
509	LOC105280171	0.037236442	uncharacterized LOC105280171
510	LOC105284746	0.037236442	negative elongation factor B
511	LOC105275435	0.03744624	alpha-(1
512	LOC105284181	0.037636133	peptidyl-prolyl cis-trans isomerase D
513	LOC105275321	0.037869617	adenosylhomocysteinase
514	LOC105275517	0.03796665	probable tubulin polyglutamylase TTLL2
515	LOC105286503	0.03796665	decaprenyl-diphosphate synthase subunit 2
516	LOC105287422	0.037977018	probable cation-transporting ATPase 13A3
517	LOC105275301	0.038090542	uncharacterized protein CG3556
518	LOC105276468	0.038090542	uncharacterized LOC105276468
519	LOC105277551	0.038090542	glycine N-methyltransferase
520	LOC105278540	0.038090542	dipeptidase 1
521	LOC105279249	0.038090542	ran GTPase-activating protein 1
522	LOC105281707	0.038090542	uncharacterized LOC105281707
523	LOC105285645	0.038090542	uncharacterized LOC105285645
524	LOC105287548	0.038090542	uncharacterized LOC105287548
525	LOC105287790	0.038090542	uncharacterized LOC105287790
526	LOC105282864	0.038322843	uncharacterized LOC105282864
527	LOC105282376	0.039194352	RNA-binding protein 7
528	LOC105281784	0.039200302	transcription termination factor 2
529	LOC105284141	0.039200302	speckle-type POZ protein B
530	LOC105285823	0.039606123	elongation of very long chain fatty acids protein AAEL008004
531	LOC105284850	0.039943123	AP-3 complex subunit sigma-1
532	LOC105276858	0.040006352	transcription factor SPT20 homolog
533	LOC105279064	0.040006352	uncharacterized LOC105279064
534	LOC105285186	0.040140578	structural maintenance of chromosomes protein 6

535	LOC105287357	0.040200326	uncharacterized LOC105287357
536	LOC105274498	0.040851277	sodium- and chloride-dependent GABA transporter 1
537	LOC105276159	0.040851277	uncharacterized protein MAL13P1.304
538	LOC105287783	0.040851277	sodium channel protein Nach
539	LOC105275608	0.041004647	uncharacterized LOC105275608
540	LOC105276234	0.041004647	phospholipid phosphatase 6
541	LOC105279933	0.041004647	leucine-rich repeat-containing G-protein coupled receptor 4
542	LOC105285453	0.041434562	kinectin
543	LOC105280382	0.042129344	major facilitator superfamily domain-containing protein 6
544	LOC105283491	0.042246189	endoribonuclease Dcr-1
545	LOC105281288	0.042424317	RNA-binding protein squid
546	LOC105282239	0.042484526	uncharacterized LOC105282239
547	LOC105281920	0.042763701	
548	LOC105287196	0.042763701	leucine-rich repeat protein 1
549	LOC105277627	0.042854837	nucleolar protein 14 homolog
550	LOC105281729	0.042921014	uncharacterized LOC105281729
551	LOC105277339	0.043338805	vicilin-like seed storage protein At2g18540
552	LOC105277777	0.043338805	pseudouridylate synthase 7 homolog
553	LOC105279952	0.043338805	uncharacterized LOC105279952
554	LOC105274595	0.043528611	chromobox protein homolog 3
555	LOC105278868	0.043615876	uncharacterized LOC105278868
556	LOC105279367	0.0436181	lipid storage droplets surface-binding protein 2
557	LOC105274633	0.043830814	ejaculatory bulb-specific protein 3
558	LOC105279164	0.043830814	uncharacterized LOC105279164
559	LOC105285169	0.043830814	sperm flagellar protein 2
560	LOC105287016	0.043833302	adenylate kinase isoenzyme 5
561	LOC105278924	0.044449161	uncharacterized LOC105278924
562	LOC105275563	0.044542688	uncharacterized LOC105275563
563	LOC105281601	0.044542688	eukaryotic translation initiation factor 4E-1A
564	LOC105287573	0.044542688	E3 ubiquitin-protein ligase RAD18
565	LOC105279259	0.044734305	ATP synthase subunit e
566	LOC105280130	0.044903873	uncharacterized LOC105280130
567	LOC105287080	0.044903873	uncharacterized LOC105287080
568	LOC105278713	0.044938377	LIM domain kinase 1
569	LOC105287888	0.045429367	uncharacterized LOC105287888
570	LOC105275551	0.045614788	uncharacterized LOC105275551
571	LOC105275867	0.045614788	uncharacterized LOC105275867
572	LOC105276573	0.045614788	leucine-rich repeat neuronal protein 2
573	LOC105278750	0.045614788	cell adhesion molecule 2
574	LOC105280293	0.045614788	pro-corazonin
575	LOC105284209	0.045614788	lysophospholipid acyltransferase 7
576	LOC105287733	0.045614788	DNA demethylase ALKBH1
577	LOC105287809	0.045614788	uncharacterized LOC105287809
578	LOC105277277	0.045746641	cytochrome P450 9e2
579	LOC105274835	0.045759955	uncharacterized LOC105274835
580	LOC105281338	0.045759955	zinc finger protein 800
581	LOC105288116	0.045759955	uncharacterized LOC105288116
582	LOC105280261	0.04596819	uncharacterized LOC105280261
583	LOC105277096	0.045989542	lysosomal Pro-X carboxypeptidase
584	LOC105281290	0.046006084	late secretory pathway protein AVL9 homolog
585	LOC105288118	0.046887354	stress-associated endoplasmic reticulum protein 2
586	LOC105284513	0.047266765	putative E3 ubiquitin-protein ligase UBR7
587	LOC105282762	0.047379082	L-lactate dehydrogenase-like
588	LOC105282263	0.048000578	uncharacterized LOC105282263
589	LOC105276691	0.048240962	E3 ubiquitin-protein ligase RNF14
590	LOC105285279	0.048240962	tachykinins
591	LOC105284709	0.048566923	uncharacterized LOC105284709
592	LOC105276523	0.048582277	acetyl-coenzyme A synthetase
593	LOC105287556	0.048940312	longitudinals lacking protein
594	LOC105280565	0.049387746	DNA-directed RNA polymerases I and III subunit RPAC1
595	LOC105281982	0.04942483	cytochrome c oxidase assembly factor 3
596	LOC105287317	0.049864337	actin
