# Supplementary material for: Clonal raider ant brain transcriptomics identifies candidate molecular mechanisms for reproductive division of labor
Source: BMC Biol. 2018 Aug 13;16:89. doi: 10.1186/s12915-018-0558-8 (PMC6090591; doi:10.1186/s12915-018-0558-8)
Supplement: Supplementary file 3 — All gene clusters identified, and their corresponding enrichment for differentially expressed genes, gene ontology terms, and transcription factor binding sites. (PDF 124 kb) [file 12915_2018_558_MOESM3_ESM.pdf]

| Column heading | Description |
|---|---|
| cluster | Cluster id. This is the id that is referenced throughout the paper. |
| change_int | The interval in which the greatest change is observed. 0: 0-12 hrs, 1: 12-24hrs, 2: 24-48 hrs, 3: 48-96 hrs. |
| change_mag | The direction and magnitude of change in gene expression at the interval indicated above. |
| num_genes | the number of genes assigned to the cluster |
| num_degs | the number of significantly differentially expressed genes (DEGs) assigned to the cluster |
| % DEGs | the percentage of DEGs in the cluster |
| odds_rat | the odds ratio from a fisher's exact test, examining enrichment of the cluster for DEGs |
| p_val | the p-value calculated from fisher's exact test for cluster enrichment of DEGs |
| q_val | the benjamini-hochberg FDR corrected p-value for DEG enrichment (yellow if <0.05) |
| # GO terms | the number of significantly enriched gene ontology terms (green if >0) |
| # TFBS | the number of significantly enriched transcription factor binding sites (green if >0) |
| GO Term | GO terms ID, class and description |
| TFBS | TFBS name (*Drosophila melanogaster* ortholog) and the FDR q-value. |

# Transition from brood care to reproduction

| | Cluster information on pattern of expression | | Cluster Enrichment for Differentially Expressed Genes | | | | | | | | | |
|---|---|---|---|---|---|---|---|---|---|---|---|---|
| cluster | change_int | change_mag | num_genes | num_degs | % DEGs | odds_rat | p_val | q_val | # GO terms | # TFBS | GO Term ID, type and definition | Transcription Factor Binding Site and q-value |
| 35 | 0 | -0.57 | 167 | 19 | 11.4% | 2.87 | 0.0001 | 0.0016 | 1 | 1 | GO:0020037 [MF] 'heme binding' GO:0016705 [MF] 'oxidoreductase activity, acting on paired donors, with incorporation or reduction of molecular oxygen' GO:0005506 [MF] 'iron ion binding' | OdsH(0.0099) exex(0.0099) CG11294(0.0144) al(0.0153) Dr(0.0157) ro(0.0157) Rx(0.0157) en(0.0173) Vsx2(0.0174) Pph13(0.0186) otp(0.0189) CG32532(0.0194) unpg(0.0194) inv(0.0217) slou(0.0224) Vsx1(0.0228) repo(0.0237) CG9876(0.0246) ind(0.0258) unc-4(0.0278) Lim1(0.0294) hbn(0.0334) ftz(0.0461) Awh(0.0473) |

| | | | | | | | | | | | | |
|---|---|---|---|---|---|---|---|---|---|---|---|---|
| 69 | 1 | -0.52 | 55 | 8 | 14.5% | 3.76 | 0.0025 | 0.0239 | 1 | 1 | GO:0043169 [MF] 'cation binding' GO:0004252 [MF] 'serine-type endopeptidase activity' GO:0052689 [MF] 'carboxylic ester hydrolase activity' GO:0016787 [MF] 'hydrolase activity' GO:0003824 [MF] 'catalytic activity' GO:0006508 [BP] 'proteolysis' GO:0007160 [BP] 'cell-matrix adhesion' GO:0009166 [BP] 'nucleotide catabolic process' | |
| 4 | 3 | 1.16 | 156 | 31 | 19.9% | 5.67 | 0 | 0 | 1 | 1 | GO:0008061 [MF] 'chitin binding' GO:0005319 [MF] 'lipid transporter activity' GO:0016705 [MF] 'oxidoreductase activity, acting on paired donors, with incorporation or reduction of molecular oxygen' GO:0005506 [MF] 'iron ion binding' GO:0005576 [CL] 'extracellular region' GO:0006030 [BP] 'chitin metabolic process' GO:0006869 [BP] 'lipid transport' | btn(0.0103) CG18599(0.0108) CG9876(0.0148) lbl(0.0148) gt(0.0152) inv(0.0160) pb(0.0160) Rx(0.0163) CG32105(0.0172) fkh(0.0172) repo(0.0173) PHDP(0.0177) zen(0.0192) zen2(0.0194) HGTX(0.0194) br(0.0194) H2.0(0.0202) Vsx2(0.0217) ro(0.0223) exex(0.0226) lms(0.0233) onecut(0.0237) slou(0.0251) slp1(0.0251) otp(0.0251) Vsx1(0.0258) Scr(0.0267) hbn(0.0272) ap(0.0272) eve(0.0272) NK7.1(0.0277) en(0.0278) Pph13(0.0278) Six4(0.0279) Lim1(0.0302) CG11617(0.0308) CG32532(0.0326) unpg(0.0348) C15(0.0359) CG4328-RA(0.0380) Lim3(0.0393) Dbx(0.0411) ems(0.0416) lab(0.0430) Dll(0.0458) Awh(0.0494) |
| 19 | 3 | 0.59 | 245 | 58 | 23.7% | 7.41 | 0 | 0 | 1 | 1 | | fkh(0.0461) |
| 21 | 3 | -0.28 | 315 | 25 | 7.9% | 1.93 | 0.003 | 0.025 | 1 | 1 | GO:0005216 [MF] 'ion channel activity' GO:0006811 [BP] 'ion transport' | opa(0.0000) Trl(0.0001) Mad(0.0002) btd(0.0003) usp(0.0013) h(0.0053) gcm2(0.0096) dl(var.2)(0.0096) brk(0.0148) Ets21C(0.0402) |
| 6 | 3 | -0.43 | 231 | 28 | 12.1% | 3.12 | 0 | 0 | 1 | 1 | | |
| 7 | 3 | -0.31 | 35 | 7 | 20.0% | 5.52 | 0.0007 | 0.0074 | 1 | 1 | | |
| 36 | 3 | 0.28 | 73 | 13 | 17.8% | 4.83 | 0 | 0.0002 | 1 | 1 | | |
| 44 | 3 | 0.27 | 353 | 41 | 11.6% | 3.01 | 0 | 0 | 1 | 1 | | |

| | | | | | | | | | | | | |
|---|---|---|---|---|---|---|---|---|---|---|---|---|
| 3 | 0 | 0.19 | 341 | 5 | 1.5% | 0.32 | 0.9993 | 1 | 1 | 1 | | Mad(0.0073) hkb(0.0173) brk(0.0223) Eip74EF(0.0415) |
| 12 | 0 | -0.08 | 319 | 11 | 3.4% | 0.78 | 0.8292 | 1 | 1 | 1 | GO:0004984 [MF] 'olfactory receptor activity' GO:0005549 [MF] 'odorant binding' GO:0007608 [BP] 'sensory perception of smell' | zen(0.0105) pb(0.0117) Dfd(0.0194) lab(0.0219) CG18599(0.0229) Scr(0.0237) vvl(0.0278) Antp(0.0278) E5(0.0348) lbe(0.0367) btn(0.0461) Lim3(0.0461) zen2(0.0475) |
| 17 | 0 | 0.04 | 481 | 13 | 2.7% | 0.6 | 0.9796 | 1 | 1 | 1 | GO:0015074 [BP] 'DNA integration' | odd(0.0162) Gsc(0.0382) oc(0.0434) |
| 26 | 0 | 0.55 | 194 | 6 | 3.1% | 0.7 | 0.8564 | 1 | 1 | 1 | | h(0.0000) Mad(0.0000) brk(0.0003) opa(0.0068) CTCF(0.0190) btd(0.0194) hkb(0.0308) usp(0.0421) |
| 27 | 0 | -0.14 | 202 | 5 | 2.5% | 0.55 | 0.9441 | 1 | 1 | 1 | GO:0006366 [BP] 'transcription from RNA polymerase II promoter' | lab(0.0118) C15(0.0157) cad(0.0173) br(var.3)(0.0212) Dbx(0.0237) Cf2(0.0277) exex(0.0278) Ubx(0.0279) Abd-B(0.0302) abd-A(0.0303) br(0.0341) hb(0.0355) vnd(0.0392) Pph13(0.0475) |
| 29 | 0 | 0.49 | 83 | 8 | 9.6% | 2.35 | 0.0283 | 0.1433 | 1 | 1 | | opa(0.0000) Mad(0.0000) usp(0.0000) Trl(0.0001) brk(0.0002) hkb(0.0003) h(0.0015) btd(0.0042) |
| 42 | 0 | 0.64 | 117 | 0 | 0.0% | 0 | 1 | 1 | 1 | 1 | | Mad(0.0002) opa(0.0017) brk(0.0108) usp(0.0411) |

| | | | | | | | | | | | | |
|---|---|---|---|---|---|---|---|---|---|---|---|---|
| 55 | 0 | -0.24 | 343 | 25 | 7.3% | 1.75 | 0.0087 | 0.059 | 1 | 1 | GO:0004518 [MF] 'nuclease activity' GO:0008152 [BP] 'metabolic process' | br(var.4)(0.0003) Abd-B(0.0004) cad(0.0031) C15(0.0039) CG4328-RA(0.0041) br(var.3)(0.0047) Dr(0.0138) Dll(0.0151) vnd(0.0153) Pph13(0.0157) fkh(0.0157) H2.0(0.0165) br(0.0173) Ubx(0.0194) CG15696-RA(0.0228) lbe(0.0237) CG9876(0.0237) PHDP(0.0238) D(0.0244) Dbx(0.0250) exd(0.0271) NK7.1(0.0299) tup(0.0303) hbn(0.0303) br(var.2)(0.0328) lms(0.0340) Hmx(0.0359) nub(0.0382) E5(0.0387) Lim3(0.0399) Cf2(0.0412) CG32105(0.0414) Rx(0.0416) B-H2(0.0461) abd-A(0.0461) ap(0.0461) hb(0.0480) |
| 58 | 0 | -0.1 | 340 | 7 | 2.1% | 0.45 | 0.9932 | 1 | 1 | 1 | | slbo(0.0092) Abd-B(0.0157) H2.0(0.0190) CG4328-RA(0.0206) Dbx(0.0207) |

| | | | | | | | | | | | | |
|---|---|---|---|---|---|---|---|---|---|---|---|---|
| 60 | 0 | -0.33 | 279 | 9 | 3.2% | 0.73 | 0.8654 | 1 | 1 | 1 | | eve(0.0041) CG18599(0.0054) vvl(0.0064) lab(0.0064) Scr(0.0064) pb(0.0064) E5(0.0096) HGTX(0.0096) ap(0.0096) Antp(0.0108) zen(0.0116) slou(0.0138) btn(0.0148) fkh(0.0152) vnd(0.0157) H2.0(0.0171) lms(0.0174) ftz(0.0189) ct(0.0189) ind(0.0194) br(0.0194) abd-A(0.0194) br(var.3)(0.0200) ems(0.0206) Vsx1(0.0207) Abd-B(0.0211) NK7.1(0.0212) br(var.2)(0.0237) lbl(0.0272) C15(0.0279) Awh(0.0299) zen2(0.0340) Dll(0.0380) Pph13(0.0382) nub(0.0402) inv(0.0405) Dfd(0.0415) lbe(0.0415) CG34031(0.0461) otp(0.0461) PHDP(0.0493) Rx(0.0493) Cf2(0.0497) |
| 61 | 0 | 0.37 | 290 | 9 | 3.1% | 0.7 | 0.8921 | 1 | 1 | 1 | | Mad(0.0001) h(0.0001) usp(0.0010) opa(0.0015) btd(0.0048) brk(0.0151) |
| 72 | 0 | 0.36 | 215 | 5 | 2.3% | 0.52 | 0.9612 | 1 | 1 | 1 | | opa(0.0000) Mad(0.0000) h(0.0001) brk(0.0003) CTCF(0.0157) hkb(0.0172) usp(0.0431) |
| 8 | 0 | -0.26 | 141 | 3 | 2.1% | 0.47 | 0.9493 | 1 | 1 | 1 | GO:0004812 [MF] 'aminoacyl-tRNA ligase activity' GO:0006418 [BP] 'tRNA aminoacylation for protein translation' | |
| 9 | 0 | -0.18 | 56 | 0 | 0.0% | 0 | 1 | 1 | 1 | 1 | | |
| 24 | 0 | -0.21 | 283 | 4 | 1.4% | 0.31 | 0.9986 | 1 | 1 | 1 | | |
| 34 | 0 | -0.24 | 135 | 8 | 5.9% | 1.38 | 0.238 | 0.8615 | 1 | 1 | | |
| 46 | 0 | -0.25 | 159 | 8 | 5.0% | 1.16 | 0.3932 | 1 | 1 | 1 | | |
| 48 | 0 | 0.11 | 148 | 2 | 1.4% | 0.3 | 0.9898 | 1 | 1 | 1 | | |
| 51 | 0 | -0.12 | 115 | 1 | 0.9% | 0.19 | 0.9943 | 1 | 1 | 1 | | |
| 52 | 0 | -0.06 | 533 | 6 | 1.1% | 0.24 | 1 | 1 | 1 | 1 | | |

| | | | | | | | | | | | | |
|---|---|---|---|---|---|---|---|---|---|---|---|---|
| 56 | 0 | 0.35 | 64 | 0 | 0.0% | 0 | 1 | 1 | 1 | 1 | GO:0008236 [MF] 'serine-type peptidase activity' GO:0008138 [MF] 'protein tyrosine/serine/threonine phosphatase activity' GO:0003735 [MF] 'structural constituent of ribosome' GO:0005840 [CL] 'ribosome' GO:0005622 [CL] 'intracellular' GO:0006412 [BP] 'translation' | |
| 59 | 0 | 0.2 | 75 | 2 | 2.7% | 0.6 | 0.8456 | 1 | 1 | 1 | GO:0003735 [MF] 'structural constituent of ribosome' GO:0005840 [CL] 'ribosome' GO:0005622 [CL] 'intracellular' GO:0006412 [BP] 'translation' | |
| 67 | 0 | 0 | 378 | 0 | 0.0% | 0 | 1 | 1 | 1 | 1 | GO:0004190 [MF] 'aspartic-type endopeptidase activity' GO:0016805 [MF] 'dipeptidase activity' GO:0004222 [MF] 'metalloendopeptidase activity' GO:0003676 [MF] 'nucleic acid binding' GO:0031012 [CL] 'extracellular matrix' GO:0015074 [BP] 'DNA integration' GO:0006508 [BP] 'proteolysis' | |
| 68 | 0 | 0.33 | 56 | 0 | 0.0% | 0 | 1 | 1 | 1 | 1 | | |
| 73 | 0 | 0.19 | 173 | 1 | 0.6% | 0.13 | 0.9996 | 1 | 1 | 1 | | |
| 74 | 0 | 0.27 | 67 | 0 | 0.0% | 0 | 1 | 1 | 1 | 1 | GO:0017048 [MF] 'Rho GTPase binding' GO:0043565 [MF] 'sequence-specific DNA binding' GO:0030036 [BP] 'actin cytoskeleton organization' GO:0006355 [BP] 'regulation of transcription, DNA-templated' GO:0006812 [BP] 'cation transport' GO:0035556 [BP] 'intracellular signal transduction' GO:0016043 [BP] 'cellular component organization' | |
| 75 | 0 | 0.13 | 131 | 2 | 1.5% | 0.34 | 0.9803 | 1 | 1 | 1 | | |
| 11 | 1 | 0.12 | 261 | 2 | 0.8% | 0.17 | 0.9999 | 1 | 1 | 1 | GO:0005488 [MF] 'binding' | cnc::maf-S(0.0237) brk(0.0475) |
| 18 | 1 | -0.11 | 315 | 2 | 0.6% | 0.14 | 1 | 1 | 1 | 1 | GO:0004984 [MF] 'olfactory receptor activity' GO:0005549 [MF] 'odorant binding' GO:0003735 [MF] 'structural constituent of ribosome' GO:0005840 [CL] 'ribosome' GO:0007608 [BP] 'sensory perception of smell' GO:0006412 [BP] 'translation' | HHEX(0.0355) H2.0(0.0356) CG4328-RA(0.0405) |

| | | | | | | | | | | | | |
|---|---|---|---|---|---|---|---|---|---|---|---|---|
| 30 | 1 | 0.15 | 282 | 13 | 4.6% | 1.06 | 0.462 | 1 | 1 | 1 | GO:0005515 [MF] 'protein binding' GO:0005802 [CL] 'trans-Golgi network' GO:0009113 [BP] 'purine nucleobase biosynthetic process' GO:0010508 [BP] 'positive regulation of autophagy' | kni(0.0157) brk(0.0233) CTCF(0.0237) h(0.0418) |
| 32 | 1 | -0.24 | 64 | 1 | 1.6% | 0.35 | 0.9431 | 1 | 1 | 1 | GO:0003735 [MF] 'structural constituent of ribosome' GO:0005840 [CL] 'ribosome' GO:0005622 [CL] 'intracellular' GO:0006412 [BP] 'translation' | fkh(0.0151) |
| 41 | 1 | 0.08 | 308 | 8 | 2.6% | 0.58 | 0.9623 | 1 | 1 | 1 | | CTCF(0.0026) h(0.0116) brk(0.0461) |
| 66 | 1 | 0.23 | 107 | 1 | 0.9% | 0.21 | 0.9918 | 1 | 1 | 1 | GO:0005337 [MF] 'nucleoside transmembrane transporter activity' | Gsc(0.0096) oc(0.0217) |
| 16 | 1 | -0.21 | 109 | 1 | 0.9% | 0.2 | 0.9925 | 1 | 1 | 1 | | |
| 25 | 1 | -0.19 | 43 | 1 | 2.3% | 0.52 | 0.854 | 1 | 1 | 1 | GO:0004984 [MF] 'olfactory receptor activity' GO:0005549 [MF] 'odorant binding' GO:0016020 [CL] 'membrane' GO:0007608 [BP] 'sensory perception of smell' | |
| 33 | 1 | -0.14 | 172 | 3 | 1.7% | 0.39 | 0.9823 | 1 | 1 | 1 | GO:0004984 [MF] 'olfactory receptor activity' GO:0005549 [MF] 'odorant binding' GO:0016020 [CL] 'membrane' GO:0007608 [BP] 'sensory perception of smell' | |
| 38 | 1 | 0.13 | 189 | 2 | 1.1% | 0.23 | 0.998 | 1 | 1 | 1 | | |
| 13 | 2 | -0.25 | 39 | 1 | 2.6% | 0.58 | 0.8253 | 1 | 1 | 1 | | gt(0.0303) CG9876(0.0303) Lim1(0.0338) exex(0.0340) lms(0.0347) inv(0.0359) en(0.0393) OdsH(0.0461) Pph13(0.0461) Awh(0.0461) unpg(0.0461) CG11294(0.0461) Rx(0.0461) repo(0.0473) Vsx2(0.0479) |
| 1 | 2 | -0.14 | 64 | 0 | 0.0% | 0 | 1 | 1 | 1 | 1 | | |
| 10 | 2 | 0.33 | 17 | 1 | 5.9% | 1.37 | 0.5323 | 1 | 1 | 1 | | |
| 14 | 2 | -0.11 | 87 | 0 | 0.0% | 0 | 1 | 1 | 1 | 1 | | |
| 40 | 2 | -0.18 | 40 | 1 | 2.5% | 0.56 | 0.833 | 1 | 1 | 1 | | |
| 43 | 2 | 0.12 | 92 | 4 | 4.3% | 0.99 | 0.5755 | 1 | 1 | 1 | | |
| 49 | 2 | 0.14 | 40 | 4 | 10.0% | 2.44 | 0.0958 | 0.4282 | 1 | 1 | | |
| 53 | 2 | 0.12 | 93 | 1 | 1.1% | 0.24 | 0.9845 | 1 | 1 | 1 | | |
| 20 | 3 | -0.11 | 204 | 5 | 2.5% | 0.55 | 0.9471 | 1 | 1 | 1 | | Eip74EF(0.0251) |

| | | | | | | | | | | | | |
|---|---|---|---|---|---|---|---|---|---|---|---|---|
| 39 | 3 | -0.19 | 391 | 14 | 3.6% | 0.81 | 0.8143 | 1 | 1 | 1 | | Mad(0.0000) opa(0.0000) btd(0.0000) brk(0.0000) gcm2(0.0021) h(0.0027) Trl(0.0056) usp(0.0092) hkb(0.0162) CTCF(0.0271) |
| 65 | 3 | -0.35 | 204 | 17 | 8.3% | 2.02 | 0.0082 | 0.059 | 1 | 1 | GO:0005249 [MF] 'voltage-gated potassium channel activity' GO:0015078 [MF] 'hydrogen ion transmembrane transporter activity' GO:0005216 [MF] 'ion channel activity' GO:0033179 [CL] 'proton-transporting V-type ATPase, V0 domain' GO:0008076 [CL] 'voltage-gated potassium channel complex' GO:0005891 [CL] 'voltage-gated calcium channel complex' GO:0055085 [BP] 'transmembrane transport' GO:0006813 [BP] 'potassium ion transport' GO:0006811 [BP] 'ion transport' GO:0051260 [BP] 'protein homooligomerization' | Mad(0.0000) opa(0.0000) btd(0.0000) brk(0.0000) Trl(0.0000) hkb(0.0000) usp(0.0004) h(0.0006) CTCF(0.0192) |
| 71 | 3 | 0.13 | 314 | 14 | 4.5% | 1.02 | 0.5082 | 1 | 1 | 1 | GO:0001104 [MF] 'RNA polymerase II transcription cofactor activity' GO:0004812 [MF] 'aminoacyl-tRNA ligase activity' GO:0004817 [MF] 'cysteine-tRNA ligase activity' GO:0051082 [MF] 'unfolded protein binding' GO:0005524 [MF] 'ATP binding' GO:0004828 [MF] 'serine-tRNA ligase activity' GO:0031072 [MF] 'heat shock protein binding' GO:0000166 [MF] 'nucleotide binding' GO:0016592 [CL] 'mediator complex' GO:0006281 [BP] 'DNA repair' GO:0009408 [BP] 'response to heat' GO:0006423 [BP] 'cysteinyl-tRNA aminoacylation' GO:0006418 [BP] 'tRNA aminoacylation for protein translation' GO:0006370 [BP] '7-methylguanosine mRNA capping' GO:0006434 [BP] 'seryl-tRNA aminoacylation' | cad(0.0334) hb(0.0461) |
| 2 | 3 | 0.11 | 178 | 6 | 3.4% | 0.76 | 0.7955 | 1 | 1 | 1 | | |
| 5 | 3 | -0.27 | 42 | 6 | 14.3% | 3.67 | 0.0093 | 0.059 | 1 | 1 | | |

| | | | | | | | | | | | | | |
|---|---|---|---|---|---|---|---|---|---|---|---|---|---|
| 15 | 3 | 0.84 | 78 | 6 | 7.7% | 1.83 | 0.1253 | 0.5014 | 1 | 1 | GO:0031177 [MF] 'phosphopantetheine binding' GO:0016740 [MF] 'transferase activity' GO:0016491 [MF] 'oxidoreductase activity' GO:0016705 [MF] 'oxidoreductase activity, acting on paired donors, with incorporation or reduction of molecular oxygen' GO:0020037 [MF] 'heme binding' GO:0016788 [MF] 'hydrolase activity, acting on ester bonds' GO:0005506 [MF] 'iron ion binding' GO:0055114 [BP] 'oxidation-reduction process' GO:0008152 [BP] 'metabolic process' GO:0009058 [BP] 'biosynthetic process' GO:0006629 [BP] 'lipid metabolic process' | |
| 22 | 3 | 0.37 | 72 | 4 | 5.6% | 1.29 | 0.3862 | 1 | 1 | 1 | GO:0016884 [MF] 'carbon-nitrogen ligase activity, with glutamine as amido-N-donor' | |
| 23 | 3 | -0.12 | 314 | 7 | 2.2% | 0.49 | 0.9857 | 1 | 1 | 1 | | |
| 28 | 3 | -0.18 | 183 | 10 | 5.5% | 1.27 | 0.2792 | 0.9644 | 1 | 1 | | |
| 31 | 3 | 0.39 | 141 | 12 | 8.5% | 2.06 | 0.0207 | 0.1122 | 1 | 1 | | |
| 37 | 3 | 0.19 | 94 | 3 | 3.2% | 0.72 | 0.7847 | 1 | 1 | 1 | | |
| 45 | 3 | 0.2 | 134 | 11 | 8.2% | 1.98 | 0.0328 | 0.1558 | 1 | 1 | | |
| 47 | 3 | 0.17 | 29 | 0 | 0.0% | 0 | 1 | 1 | 1 | 1 | | |
| 50 | 3 | 0.12 | 204 | 13 | 6.4% | 1.5 | 0.1115 | 0.4708 | 1 | 1 | | |
| 54 | 3 | -0.13 | 150 | 7 | 4.7% | 1.07 | 0.4844 | 1 | 1 | 1 | GO:0004984 [MF] 'olfactory receptor activity' GO:0005549 [MF] 'odorant binding' GO:0007608 [BP] 'sensory perception of smell' | |
| 57 | 3 | -0.12 | 354 | 9 | 2.5% | 0.56 | 0.9747 | 1 | 1 | 1 | | |
| 62 | 3 | 0.26 | 99 | 7 | 7.1% | 1.67 | 0.142 | 0.5396 | 1 | 1 | | |
| 63 | 3 | -0.2 | 262 | 20 | 7.6% | 1.84 | 0.0111 | 0.065 | 1 | 1 | GO:0004984 [MF] 'olfactory receptor activity' GO:0005549 [MF] 'odorant binding' GO:0016020 [CL] 'membrane' GO:0007608 [BP] 'sensory perception of smell' | |
| 64 | 3 | 0.19 | 117 | 1 | 0.9% | 0.19 | 0.9948 | 1 | 1 | 1 | GO:0003735 [MF] 'structural constituent of ribosome' GO:0005840 [CL] 'ribosome' GO:0015935 [CL] 'small ribosomal subunit' GO:0005622 [CL] 'intracellular' GO:0042254 [BP] 'ribosome biogenesis' GO:0006412 [BP] 'translation' | |
| 70 | 3 | -0.14 | 72 | 1 | 1.4% | 0.31 | 0.9603 | 1 | 1 | 1 | | |

| | | | | | | | | | | | | |
|---|---|---|---|---|---|---|---|---|---|---|---|---|
| 76 | 3 | 0.06 | 334 | 7 | 2.1% | 0.46 | 0.9919 | 1 | 1 | 1 | | |

# Transition from reproduction to brood care

| | Cluster information on pattern of expression | | Cluster Enrichment for Differentially Expressed Genes | | | | | | | | | |
|---|---|---|---|---|---|---|---|---|---|---|---|---|
| cluster | change_int | change_mag | num_genes | num_degs | % DEGs | odds_rat | p_val | q_val | # GO terms | # TFBS | GO Term ID, type and definition | Transcription Factor Binding Site and q-value |
| 93 | 0 | 0.25 | 134 | 16 | 11.9% | 3.02 | 0.0002 | 0.0019 | 0 | 6 | | Mad(0.0000) h(0.0001) opa(0.0015) btd(0.0019) brk(0.0025) usp(0.0076) |
| 56 | 0 | 0.21 | 91 | 22 | 24.2% | 7.21 | 0 | 0 | 1 | 0 | GO:0005789 [CL] 'endoplasmic reticulum membrane' | |
| 65 | 0 | -1.21 | 76 | 17 | 22.4% | 6.46 | 0 | 0 | 3 | 0 | GO:0005576 [CL] 'extracellular region' GO:0005615 [CL] 'extracellular space' GO:0035176 [BP] 'social behavior' | |
| 34 | 0 | 0.21 | 119 | 17 | 14.3% | 3.73 | 0 | 0.0001 | 0 | 0 | | |
| 12 | 0 | 0.48 | 82 | 13 | 15.9% | 4.19 | 0.0001 | 0.0004 | 0 | 0 | | |
| 8 | 1 | -0.35 | 134 | 48 | 35.8% | 13.2 | 0 | 0 | 0 | 1 | | fkh(0.0252) |
| 47 | 1 | -0.18 | 206 | 59 | 28.6% | 9.64 | 0 | 0 | 1 | 0 | GO:0008237 [MF] 'metallopeptidase activity' | |
| 96 | 1 | -0.26 | 109 | 17 | 15.6% | 4.13 | 0 | 0 | 0 | 0 | | |
| 90 | 2 | 0.16 | 151 | 30 | 19.9% | 5.66 | 0 | 0 | 5 | 6 | GO:0046933 [MF] 'proton-transporting ATP synthase activity, rotational mechanism' GO:0005834 [CL] 'heterotrimeric G-protein complex' GO:0046034 [BP] 'ATP metabolic process' GO:0015986 [BP] 'ATP synthesis coupled proton transport' GO:0035556 [BP] 'intracellular signal transduction' | opa(0.0000) btd(0.0001) h(0.0026) Mad(0.0029) usp(0.0117) hkb(0.0450) |
| 70 | 2 | 0.22 | 162 | 45 | 27.8% | 9.02 | 0 | 0 | 1 | 4 | GO:0055114 [BP] 'oxidation-reduction process' | Mad(0.0002) usp(0.0345) opa(0.0398) Trl(0.0475) |
| 58 | 3 | 0.25 | 126 | 15 | 11.9% | 3.01 | 0.0004 | 0.0029 | 1 | 0 | GO:0043565 [MF] 'sequence-specific DNA binding' | |
| 82 | 3 | 0.4 | 133 | 14 | 10.5% | 2.61 | 0.002 | 0.0137 | 1 | 0 | GO:0052689 [MF] 'carboxylic ester hydrolase activity' | |
| 73 | 3 | 0.66 | 72 | 23 | 31.9% | 10.7 | 0 | 0 | 0 | 0 | | |
| 91 | 3 | -0.23 | 169 | 41 | 24.3% | 7.45 | 0 | 0 | 0 | 0 | | |

| | | | | | | | | | | | | |
|---|---|---|---|---|---|---|---|---|---|---|---|---|
| 15 | 0 | 0.17 | 187 | 4 | 2.1% | 0.47 | 0.966 | 1 | 1 | 8 | GO:0005856 [CL] 'cytoskeleton' | opa(0.0013) Mad(0.0073) btd(0.0117) gcm2(0.0162) Trl(0.0162) usp(0.0209) brk(0.0450) schlank(0.0496) |
| 87 | 0 | 0.26 | 180 | 5 | 2.8% | 0.62 | 0.8991 | 1 | 4 | 2 | GO:0004889 [MF] 'acetylcholine-activated cation-selective channel activity' GO:0004129 [MF] 'cytochrome-c oxidase activity' GO:0045211 [CL] 'postsynaptic membrane' GO:0043044 [BP] 'ATP-dependent chromatin remodeling' | h(0.0073) Mad(0.0142) |
| 5 | 0 | 0 | 385 | 0 | 0.0% | 0 | 1 | 1 | 10 | 1 | GO:0004190 [MF] 'aspartic-type endopeptidase activity' GO:0003887 [MF] 'DNA-directed DNA polymerase activity' GO:0016805 [MF] 'dipeptidase activity' GO:0008408 [MF] '3'-5' exonuclease activity' GO:0008237 [MF] 'metallopeptidase activity' GO:0004222 [MF] 'metalloendopeptidase activity' GO:0003676 [MF] 'nucleic acid binding' GO:0031012 [CL] 'extracellular matrix' GO:0015074 [BP] 'DNA integration' GO:0006508 [BP] 'proteolysis' | usp(0.0373) |
| 30 | 0 | 0.22 | 545 | 2 | 0.4% | 0.08 | 1 | 1 | 0 | 3 | | h(0.0252) vis(0.0252) brk(0.0252) |
| 25 | 0 | 0.08 | 235 | 1 | 0.4% | 0.09 | 1 | 1 | 0 | 1 | | kni(0.0252) |
| 9 | 0 | -0.29 | 78 | 0 | 0.0% | 0 | 1 | 1 | 0 | 1 | | nub(0.0249) |
| 68 | 0 | -0.22 | 206 | 0 | 0.0% | 0 | 1 | 1 | 7 | 0 | GO:0004984 [MF] 'olfactory receptor activity' GO:0003743 [MF] 'translation initiation factor activity' GO:0005549 [MF] 'odorant binding' GO:0004803 [MF] 'transposase activity' GO:0007608 [BP] 'sensory perception of smell' GO:0006313 [BP] 'transposition, DNA-mediated' GO:0050909 [BP] 'sensory perception of taste' | |
| 33 | 0 | 0.12 | 255 | 1 | 0.4% | 0.08 | 1 | 1 | 3 | 0 | GO:0008479 [MF] 'queuine tRNA-ribosyltransferase activity' GO:0046873 [MF] 'metal ion transmembrane transporter activity' GO:0008616 [BP] 'queuosine biosynthetic process' | |
| 42 | 0 | -0.36 | 20 | 0 | 0.0% | 0 | 1 | 1 | 3 | 0 | GO:0004984 [MF] 'olfactory receptor activity' GO:0005549 [MF] 'odorant binding' GO:0007608 [BP] 'sensory perception of smell' | |

| | | | | | | | | | | | | |
|---|---|---|---|---|---|---|---|---|---|---|---|---|
| 89 | 0 | 0.34 | 51 | 3 | 5.9% | 1.37 | 0.3863 | 1 | 3 | 0 | GO:0005328 [MF] 'neurotransmitter:sodium symporter activity' GO:0016021 [CL] 'integral component of membrane' GO:0006836 [BP] 'neurotransmitter transport' | |
| 85 | 0 | -1.16 | 132 | 12 | 9.1% | 2.21 | 0.0129 | 0.0814 | 7 | 0 | GO:0043169 [MF] 'cation binding' GO:0004252 [MF] 'serine-type endopeptidase activity' GO:0004132 [MF] 'dCMP deaminase activity' GO:0016787 [MF] 'hydrolase activity' GO:0003824 [MF] 'catalytic activity' GO:0006220 [BP] 'pyrimidine nucleotide metabolic process' GO:0006508 [BP] 'proteolysis' | |
| 37 | 0 | -0.53 | 119 | 3 | 2.5% | 0.56 | 0.8975 | 1 | 6 | 0 | GO:0016740 [MF] 'transferase activity' GO:0016788 [MF] 'hydrolase activity, acting on ester bonds' GO:0016491 [MF] 'oxidoreductase activity' GO:0055114 [BP] 'oxidation-reduction process' GO:0008152 [BP] 'metabolic process' GO:0019441 [BP] 'tryptophan catabolic process to kynurenine' | |
| 26 | 0 | -0.23 | 157 | 4 | 2.5% | 0.57 | 0.9167 | 1 | 5 | 0 | GO:0003735 [MF] 'structural constituent of ribosome' GO:0005840 [CL] 'ribosome' GO:0015935 [CL] 'small ribosomal subunit' GO:0042254 [BP] 'ribosome biogenesis' GO:0006412 [BP] 'translation' | |
| 61 | 0 | -0.52 | 29 | 2 | 6.9% | 1.62 | 0.3637 | 1 | 1 | 0 | GO:0005215 [MF] 'transporter activity' | |
| 80 | 0 | -0.19 | 95 | 0 | 0.0% | 0 | 1 | 1 | 1 | 0 | GO:0006850 [BP] 'mitochondrial pyruvate transport' | |
| 1 | 0 | 0.22 | 66 | 1 | 1.5% | 0.34 | 0.948 | 1 | 0 | 0 | | |
| 3 | 0 | -0.39 | 132 | 0 | 0.0% | 0 | 1 | 1 | 0 | 0 | | |
| 4 | 0 | 0.25 | 214 | 2 | 0.9% | 0.2 | 0.9993 | 1 | 0 | 0 | | |
| 17 | 0 | 0.17 | 286 | 2 | 0.7% | 0.15 | 1 | 1 | 0 | 0 | | |
| 40 | 0 | 0.44 | 82 | 0 | 0.0% | 0 | 1 | 1 | 0 | 0 | | |
| 41 | 0 | -0.15 | 192 | 3 | 1.6% | 0.34 | 0.9913 | 1 | 0 | 0 | | |
| 48 | 0 | 0.45 | 214 | 2 | 0.9% | 0.2 | 0.9993 | 1 | 0 | 0 | | |
| 51 | 0 | 0.16 | 143 | 0 | 0.0% | 0 | 1 | 1 | 0 | 0 | | |
| 52 | 0 | -0.18 | 246 | 3 | 1.2% | 0.27 | 0.9988 | 1 | 0 | 0 | | |
| 63 | 0 | -0.24 | 113 | 6 | 5.3% | 1.23 | 0.3727 | 1 | 0 | 0 | | |
| 74 | 0 | -0.19 | 149 | 3 | 2.0% | 0.45 | 0.9611 | 1 | 0 | 0 | | |
| 78 | 0 | -0.59 | 183 | 10 | 5.5% | 1.27 | 0.2792 | 1 | 0 | 0 | | |
| 84 | 0 | -0.29 | 159 | 0 | 0.0% | 0 | 1 | 1 | 0 | 0 | | |

| | | | | | | | | | | | | |
|---|---|---|---|---|---|---|---|---|---|---|---|---|
| 88 | 0 | -0.32 | 309 | 10 | 3.2% | 0.73 | 0.8733 | 1 | 0 | 0 | | |
| 95 | 0 | -0.09 | 662 | 3 | 0.5% | 0.1 | 1 | 1 | 0 | 0 | | |
| 32 | 0 | -0.1 | 309 | 13 | 4.2% | 0.96 | 0.5962 | 1 | 0 | 0 | | |
| 21 | 0 | -0.16 | 130 | 0 | 0.0% | 0 | 1 | 1 | 0 | 0 | | |
| 62 | 0 | -0.33 | 33 | 0 | 0.0% | 0 | 1 | 1 | 0 | 0 | | |
| 19 | 1 | 0.25 | 47 | 0 | 0.0% | 0 | 1 | 1 | 2 | 0 | GO:0008146 [MF] 'sulfotransferase activity' GO:0005783 [CL] 'endoplasmic reticulum' | |
| 92 | 1 | -0.18 | 56 | 1 | 1.8% | 0.4 | 0.9185 | 1 | 4 | 0 | GO:0004984 [MF] 'olfactory receptor activity' GO:0005549 [MF] 'odorant binding' GO:0016020 [CL] 'membrane' GO:0007608 [BP] 'sensory perception of smell' | |
| 2 | 1 | -0.25 | 27 | 0 | 0.0% | 0 | 1 | 1 | 7 | 0 | GO:0003995 [MF] 'acyl-CoA dehydrogenase activity' GO:0016627 [MF] 'oxidoreductase activity, acting on the CH-CH group of donors' GO:0050660 [MF] 'flavin adenine dinucleotide binding' GO:0006635 [BP] 'fatty acid beta-oxidation' GO:0006631 [BP] 'fatty acid metabolic process' GO:0055114 [BP] 'oxidation-reduction process' GO:0008152 [BP] 'metabolic process' | |
| 53 | 1 | -0.19 | 17 | 1 | 5.9% | 1.37 | 0.5323 | 1 | 1 | 0 | GO:0003824 [MF] 'catalytic activity' | |
| 64 | 1 | -0.11 | 159 | 8 | 5.0% | 1.16 | 0.3932 | 1 | 0 | 0 | | |
| 86 | 1 | -0.33 | 49 | 5 | 10.2% | 2.5 | 0.0616 | 0.3254 | 0 | 0 | | |
| 13 | 1 | 0.3 | 4 | 0 | 0.0% | 0 | 1 | 1 | 0 | 0 | | |
| 24 | 1 | -0.18 | 73 | 0 | 0.0% | 0 | 1 | 1 | 0 | 0 | | |
| 29 | 1 | -0.17 | 130 | 3 | 2.3% | 0.51 | 0.9275 | 1 | 0 | 0 | | |
| 83 | 1 | -0.18 | 46 | 0 | 0.0% | 0 | 1 | 1 | 0 | 0 | | |
| 69 | 2 | 0.36 | 20 | 1 | 5.0% | 1.15 | 0.5911 | 1 | 2 | 0 | GO:0004553 [MF] 'hydrolase activity, hydrolyzing O-glycosyl compounds' GO:0005975 [BP] 'carbohydrate metabolic process' | |
| 20 | 2 | 0.31 | 34 | 1 | 2.9% | 0.66 | 0.7815 | 1 | 1 | 0 | GO:0019752 [BP] 'carboxylic acid metabolic process' | |
| 35 | 2 | 0.14 | 179 | 1 | 0.6% | 0.12 | 0.9997 | 1 | 1 | 0 | GO:0000166 [MF] 'nucleotide binding' | |
| 44 | 2 | 0.24 | 80 | 2 | 2.5% | 0.56 | 0.8702 | 1 | 4 | 0 | GO:0003777 [MF] 'microtubule motor activity' GO:0005198 [MF] 'structural molecule activity' GO:0006511 [BP] 'ubiquitin-dependent protein catabolic process' GO:0007018 [BP] 'microtubule-based movement' | |

| | | | | | | | | | | | | |
|---|---|---|---|---|---|---|---|---|---|---|---|---|
| 16 | 2 | 0.1 | 209 | 7 | 3.3% | 0.76 | 0.8132 | 1 | 2 | 0 | GO:0015991 [BP] 'ATP hydrolysis coupled proton transport' GO:0033180 [CL] 'proton-transporting V-type ATPase, V1 domain' | |
| 18 | 2 | -0.09 | 140 | 7 | 5.0% | 1.15 | 0.413 | 1 | 1 | 0 | GO:0016972 [MF] 'thiol oxidase activity' | |
| 23 | 2 | 0.46 | 25 | 0 | 0.0% | 0 | 1 | 1 | 2 | 0 | GO:0008061 [MF] 'chitin binding' GO:0006030 [BP] 'chitin metabolic process' | |
| 46 | 2 | 0.12 | 112 | 8 | 7.1% | 1.69 | 0.1169 | 0.5553 | 0 | 0 | | |
| 28 | 2 | 0.1 | 168 | 9 | 5.4% | 1.24 | 0.3136 | 1 | 0 | 0 | | |
| 31 | 2 | 0.17 | 113 | 0 | 0.0% | 0 | 1 | 1 | 0 | 0 | | |
| 36 | 2 | 0.07 | 231 | 2 | 0.9% | 0.19 | 0.9996 | 1 | 0 | 0 | | |
| 59 | 2 | -0.17 | 33 | 2 | 6.1% | 1.41 | 0.4261 | 1 | 0 | 0 | | |
| 22 | 2 | 0.23 | 36 | 0 | 0.0% | 0 | 1 | 1 | 0 | 0 | | |
| 27 | 2 | -0.08 | 117 | 1 | 0.9% | 0.19 | 0.9948 | 1 | 0 | 0 | | |
| 43 | 2 | -0.21 | 21 | 0 | 0.0% | 0 | 1 | 1 | 0 | 0 | | |
| 49 | 2 | -0.13 | 120 | 0 | 0.0% | 0 | 1 | 1 | 0 | 0 | | |
| 60 | 2 | 0.4 | 16 | 0 | 0.0% | 0 | 1 | 1 | 0 | 0 | | |
| 77 | 3 | 0.36 | 65 | 0 | 0.0% | 0 | 1 | 1 | 10 | 1 | GO:0016641 [MF] 'oxidoreductase activity, acting on the CH-NH2 group of donors, oxygen as acceptor' GO:0004930 [MF] 'G-protein coupled receptor activity' GO:0020037 [MF] 'heme binding' GO:0000062 [MF] 'fatty-acyl-CoA binding' GO:0003824 [MF] 'catalytic activity' GO:0016705 [MF] 'oxidoreductase activity, acting on paired donors, with incorporation or reduction of molecular oxygen' GO:0005506 [MF] 'iron ion binding' GO:0055114 [BP] 'oxidation-reduction process' GO:0008152 [BP] 'metabolic process' GO:0008299 [BP] 'isoprenoid biosynthetic process' | . |
| 10 | 3 | -0.2 | 84 | 8 | 9.5% | 2.32 | 0.0301 | 0.1788 | 0 | 3 | | opa(0.0064) btd(0.0099) Mad(0.0290) |
| 50 | 3 | 0.13 | 129 | 0 | 0.0% | 0 | 1 | 1 | 4 | 0 | GO:0004984 [MF] 'olfactory receptor activity' GO:0005549 [MF] 'odorant binding' GO:0016020 [CL] 'membrane' GO:0007608 [BP] 'sensory perception of smell' | |
| 45 | 3 | 0.09 | 145 | 1 | 0.7% | 0.15 | 0.9985 | 1 | 3 | 0 | GO:0004984 [MF] 'olfactory receptor activity' GO:0005549 [MF] 'odorant binding' GO:0007608 [BP] 'sensory perception of smell' | |

| | | | | | | | | | | | | |
|---|---|---|---|---|---|---|---|---|---|---|---|---|
| 94 | 3 | 0.82 | 135 | 10 | 7.4% | 1.76 | 0.0716 | 0.3581 | 8 | 0 | GO:0020037 [MF] 'heme binding' GO:0031177 [MF] 'phosphopantetheine binding' GO:0016491 [MF] 'oxidoreductase activity' GO:0016705 [MF] 'oxidoreductase activity, acting on paired donors, with incorporation or reduction of molecular oxygen' GO:0005215 [MF] 'transporter activity' GO:0005506 [MF] 'iron ion binding' GO:0055114 [BP] 'oxidation-reduction process' GO:0008152 [BP] 'metabolic process' | |
| 66 | 3 | -0.08 | 173 | 2 | 1.2% | 0.25 | 0.9962 | 1 | 3 | 0 | GO:0005248 [MF] 'voltage-gated sodium channel activity' GO:0001518 [CL] 'voltage-gated sodium channel complex' GO:0016012 [CL] 'sarcoglycan complex' | |
| 11 | 3 | 0.29 | 43 | 0 | 0.0% | 0 | 1 | 1 | 0 | 0 | | |
| 14 | 3 | -0.08 | 175 | 2 | 1.1% | 0.25 | 0.9965 | 1 | 0 | 0 | | |
| 39 | 3 | 0.06 | 291 | 0 | 0.0% | 0 | 1 | 1 | 0 | 0 | | |
| 54 | 3 | 0.22 | 128 | 5 | 3.9% | 0.89 | 0.6633 | 1 | 0 | 0 | | |
| 57 | 3 | 0.2 | 140 | 2 | 1.4% | 0.31 | 0.9861 | 1 | 0 | 0 | | |
| 67 | 3 | 0.14 | 282 | 1 | 0.4% | 0.08 | 1 | 1 | 0 | 0 | | |
| 72 | 3 | -0.03 | 563 | 0 | 0.0% | 0 | 1 | 1 | 0 | 0 | | |
| 79 | 3 | -0.13 | 269 | 11 | 4.1% | 0.93 | 0.6326 | 1 | 0 | 0 | | |
| 38 | 3 | 0.22 | 56 | 1 | 1.8% | 0.4 | 0.9185 | 1 | 0 | 0 | | |
| 55 | 3 | -0.22 | 204 | 15 | 7.4% | 1.76 | 0.0342 | 0.191 | 0 | 0 | | |
| 6 | 3 | -0.47 | 15 | 1 | 6.7% | 1.56 | 0.4886 | 1 | 0 | 0 | | |
| 7 | 3 | -0.17 | 118 | 1 | 0.8% | 0.19 | 0.995 | 1 | 0 | 0 | | |
| 71 | 3 | -0.12 | 185 | 3 | 1.6% | 0.36 | 0.9888 | 1 | 0 | 0 | | |
| 76 | 3 | 0.25 | 47 | 1 | 2.1% | 0.47 | 0.878 | 1 | 0 | 0 | | |
| 81 | 3 | -0.27 | 71 | 1 | 1.4% | 0.31 | 0.9584 | 1 | 0 | 0 | | |
