# Supplementary material for: Clonal raider ant brain transcriptomics identifies candidate molecular mechanisms for reproductive division of labor
Source: BMC Biol. 2018 Aug 13;16:89. doi: 10.1186/s12915-018-0558-8 (PMC6090591; doi:10.1186/s12915-018-0558-8)
Supplement: Supplementary file 5 — Summary of clusters enriched for DEGs. (PDF 29 kb) [file 12915_2018_558_MOESM5_ESM.pdf]

Summary of clusters enriched for DEGs.

Time interval	Number of genes in clusters (enriched for DEGs) with maximal change in expression for each time interval	Proportion of genes in clusters (enriched for DEGs) with maximal change in expression for each time interval (%)	Number of DEGs in clusters (enriched for DEGs) with maximal change in expression for each time interval	Proportion of DEGs in clusters (enriched for DEGs) with maximal change in expression for each time interval (%)	Ovary score
Reproduction to brood care					
0-12 hrs	502	28.5	85	22.5	3.4
12-24 hrs	449	25.5	124	32.9	3.15
24-48 hrs	313	17.7	75	19.9	1.95
48-96 hrs	500	28.3	93	24.7	1.7
Brood care to reproduction					
0-12 hrs	167	10.2	19	8.3	1.3
12-24 hrs	55	3.4	8	3.5	1.25
24-48 hrs	0	0	0	0	1.75
48-96 hrs	1408	86.4	203	88.3	2.95
