# Supplementary material for: Clonal raider ant brain transcriptomics identifies candidate molecular mechanisms for reproductive division of labor
Source: BMC Biol. 2018 Aug 13;16:89. doi: 10.1186/s12915-018-0558-8 (PMC6090591; doi:10.1186/s12915-018-0558-8)
Supplement: Supplementary file 6 — Basic network statistics of gene clusters for time course gene expression between both reproductive and brood care phase transitions. (PDF 51 kb) [file 12915_2018_558_MOESM6_ESM.pdf]

Basic network statistics of gene clusters for time course gene expression between both reproductive and brood care phase transitions.

Network parameters	Complete network	Conserved network
Number of nodes	172	143
Connected components	2	11
Network Diameter	4	10
Network Centralization	0.203	0.073
Average number of neighbors	40.744	1.846
Network density	0.238	0.013
Network heterogeneity	0.389	0.863
Isolated nodes	1	0

Each gene was assigned to a cluster based on its expression profile. The complete network was constructed using the clusters from both phase transitions. The network edges represent the number of genes shared between two clusters from different transitions. No cluster will share genes with any other cluster from the same transition, and each gene is represented on one and only one edge. The conserved network is a subset of the complete network, containing all clusters that share more genes than expected by chance (Jaccard Index, 1000 permutations, q-value ≤ 0.05).

Network statistics were calculated in Cytoscape v.3.5.0. Definitions for the terms as used by Cytoscape are described in http://med.bioinf.mpi-inf.mpg.de/netanalyzer/help/2.7/index.html#simple.

Number of nodes: The number of gene clusters in the network. Each node represents a single gene cluster from the either brood care to reproduction transition or the reproduction to brood care transition.
Connected components: The number of groups of pairwise-connected nodes within the network. Fewer connected components suggests stronger connectivity.
Network Diameter: The largest number of edges necessary to connect any two (connected) nodes in the network.
Network Centralization: The tendency for certain nodes to be more central than others in the network. Values range from 0 (a fully decentralized network) to 1 (a fully-centralized network).
Average number of neighbors: The average number of nodes each node is immediately connected with.
Network density: The normalized measure of average number of neighbors, which indicates the density of edges within a network. Values range from 0 (no edges) to 1 (a clique).
Network heterogeneity: The tendency of the network to contain hub nodes. Values range between 0 (no hubs) to 1.
Isolated nodes: The number of nodes that are not connected to any other nodes.
