# Supplementary material for: Clonal raider ant brain transcriptomics identifies candidate molecular mechanisms for reproductive division of labor
Source: BMC Biol. 2018 Aug 13;16:89. doi: 10.1186/s12915-018-0558-8 (PMC6090591; doi:10.1186/s12915-018-0558-8)
Supplement: Supplementary file 7 — GO terms significantly enriched in clusters enriched for DEGs. Only two of these GO terms were common to both transitions (RB: transition from reproduction to brood care; BR: transition from brood care to reproduction). The diameter of the circles is proportional to the number of enriched GO terms. (PDF 97 kb) [file 12915_2018_558_MOESM7_ESM.pdf]

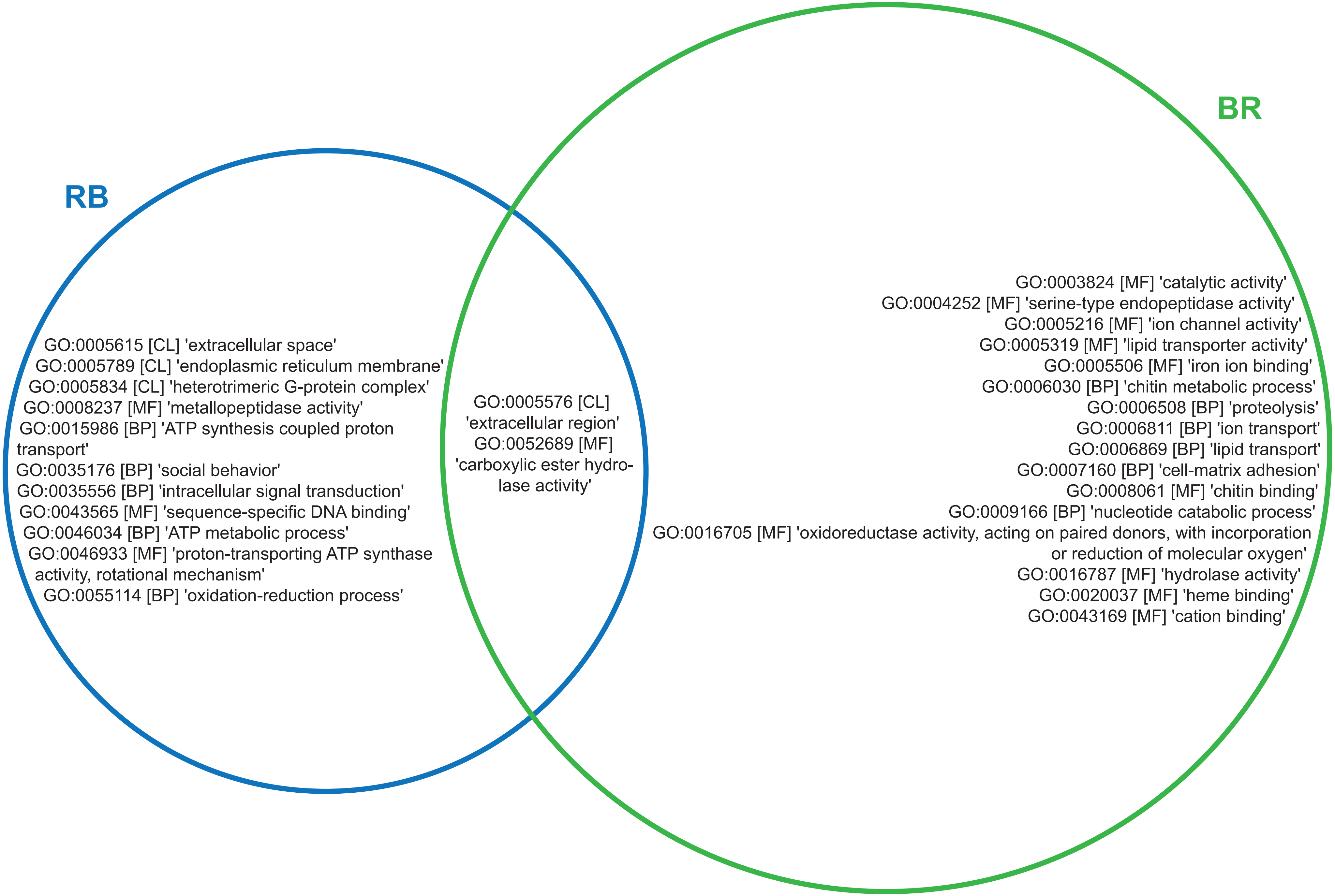
RB
BR
GO:0005615 [CL] 'extracellular space'
GO:0005789 [CL] 'endoplasmic reticulum membrane'
GO:0005834 [CL] 'heterotrimeric G-protein complex'
GO:0008237 [MF] 'metallopeptidase activity'
GO:0015986 [BP] 'ATP synthesis coupled proton transport'
GO:0035176 [BP] 'social behavior'
GO:0035556 [BP] 'intracellular signal transduction'
GO:0043565 [MF] 'sequence-specific DNA binding'
GO:0046034 [BP] 'ATP metabolic process'
GO:0046933 [MF] 'proton-transporting ATP synthase activity, rotational mechanism'
GO:0055114 [BP] 'oxidation-reduction process'
GO:0005576 [CL] 'extracellular region'
GO:0052689 [MF] 'carboxylic ester hydrolase activity'
GO:0003824 [MF] 'catalytic activity'
GO:0004252 [MF] 'serine-type endopeptidase activity'
GO:0005216 [MF] 'ion channel activity'
GO:0005319 [MF] 'lipid transporter activity'
GO:0005506 [MF] 'iron ion binding'
GO:0006030 [BP] 'chitin metabolic process'
GO:0006508 [BP] 'proteolysis'
GO:0006811 [BP] 'ion transport'
GO:0006869 [BP] 'lipid transport'
GO:0007160 [BP] 'cell-matrix adhesion'
GO:0008061 [MF] 'chitin binding'
GO:0009166 [BP] 'nucleotide catabolic process'
GO:0016705 [MF] 'oxidoreductase activity, acting on paired donors, with incorporation or reduction of molecular oxygen'
GO:0016787 [MF] 'hydrolase activity'
GO:0020037 [MF] 'heme binding'
GO:0043169 [MF] 'cation binding'
