# Supplementary material for: Clonal raider ant brain transcriptomics identifies candidate molecular mechanisms for reproductive division of labor
Source: BMC Biol. 2018 Aug 13;16:89. doi: 10.1186/s12915-018-0558-8 (PMC6090591; doi:10.1186/s12915-018-0558-8)
Supplement: Supplementary file 9 — Top 40 DEGs (ranked according to log2 fold change in expression for control vs 12-h time point and control vs 96-h time point for each transition). (PDF 50 kb) [file 12915_2018_558_MOESM9_ESM.pdf]

	Top 40 DEGs (highest log2 fold change in expression)							
	Reproduction to brood care				Brood care to reproduction			
	0-96 hours		0-12 hours		0-96 hours		0-12 hours	
	Gene	Annotation	Gene	Annotation	Gene	Annotation	Gene	Annotation
1	LOC105286381	uncharacterized LOC105286381	LOC105285921	uncharacterized LOC105285921	LOC105281428_Q	queen Vg	LOC105287093	uncharacterized LOC105287093
2	LOC105285921	uncharacterized LOC105285921	LOC105286381	uncharacterized LOC105286381	LOC105277525	uncharacterized LOC105277525	LOC105276678	transferrin
3	LOC105285920	vegetative cell wall protein gp1	LOC105276678	transferrin	LOC105279933	leucine-rich repeat-containing G-protein coupled receptor 4	LOC105278524	LIRP - ILP2
4	LOC105275344	csp8	LOC105285823	elongation of very long chain fatty acids protein	LOC105277435	targeting protein for Xklp2	LOC105278931	venom allergen 3
5	LOC105276678	transferrin	LOC105278724	fatty acid synthase	LOC105287193	uncharacterized LOC105287193	LOC105277716	facilitated trehalose transporter Tret1
6	LOC105281428_Q	queen Vg	LOC105275344	csp8	LOC105281920	aminopeptidase N-like	LOC105282359	myosin regulatory light chain 2
7	LOC105275345	ejaculatory bulb-specific protein 3	LOC105275345	ejaculatory bulb-specific protein 3	LOC105279314	trypsin inhibitor	LOC105283737	myosin heavy chain, muscle
8	LOC105285823	elongation of very long chain fatty acids protein	LOC105287552	snakeskin protein	LOC105285920	vegetative cell wall protein gp1	LOC105281707	uncharacterized LOC105281707
9	LOC105277525	uncharacterized LOC105277525	LOC105277525	uncharacterized LOC105277525	LOC105284141	speckle-type POZ protein B	LOC105287552	snakeskin protein
10	LOC105287552	snakeskin protein	LOC105279933	leucine-rich repeat-containing G-protein coupled receptor 4	LOC105282036	G2/mitotic-specific cyclin-B	LOC105278758	actin
11	LOC105276573	leucine-rich repeat neuronal protein 2	LOC105276573	leucine-rich repeat neuronal protein 2	LOC105287714	protein hunchback	LOC105276573	leucine-rich repeat neuronal protein 2
12	LOC105278724	fatty acid synthase	LOC105283737	myosin heavy chain, muscle	LOC105281786	hyaluronan mediated motility receptor	LOC105281721	uncharacterized LOC105281721
13	LOC105281012	uncharacterized LOC105281012	LOC105276433	box A-binding factor	LOC105284119	ribonuclease H-like	LOC105281786	hyaluronan mediated motility receptor
14	LOC105281708	uncharacterized LOC105281708	LOC105277700	actin, clone 205	LOC105281784	transcription termination factor 2	LOC105277700	actin, clone 205
15	LOC105288201	uncharacterized LOC105288201	LOC105277377	troponin C	LOC105288201	uncharacterized LOC105288201	LOC105277377	troponin C
16	LOC105284141	speckle-type POZ protein B	LOC105282359	myosin regulatory light chain 2	LOC105287010	uncharacterized LOC105287010	LOC105275507	PDZ and LIM domain protein 3
17	LOC105282036	G2/mitotic-specific cyclin-B	LOC105287093	uncharacterized LOC105287093	LOC105283509	zinc finger BED domain-containing protein 1-like	LOC105279933	leucine-rich repeat-containing G-protein coupled receptor 4
18	LOC105284181	peptidyl-prolyl cis-trans isomerase D	LOC105287812	uncharacterized LOC105287812	LOC105279586	uncharacterized LOC105279586	LOC105277728	uncharacterized LOC105277728
19	LOC105287714	protein hunchback	LOC105281721	uncharacterized LOC105281721	LOC105284633	gliomedin	LOC105280772	uncharacterized LOC105280772
20	LOC105277435	targeting protein for Xklp2	LOC105285936	glutathione S-transferase 1-1	LOC105287013	kinesin-like protein KIF20B	LOC105279907	uncharacterized LOC105279907
21	LOC105276433	box A-binding factor	LOC105278758	actin	LOC105283233	fibrillin-1	LOC105279559	glycine receptor subunit alpha-2-like
22	LOC105275236	carboxypeptidase M	LOC105281449	muscle M-line assembly protein unc-89-like	LOC105283266	lymphoid-specific helicase	LOC105283796	ornithine decarboxylase 2-like
23	LOC105287193	uncharacterized LOC105287193	LOC105280202	glycine-rich cell wall structural protein	LOC105278524	LIRP - ILP2	LOC105285920	vegetative cell wall protein gp1
24	LOC105281920	aminopeptidase N-like	LOC105275821	dnaJ homolog subfamily C member 22	LOC105283796	ornithine decarboxylase 2-like	LOC105276699	uncharacterized LOC105276699
25	LOC105283615	serine protease nudel	LOC105287204	twitchin	LOC105283366	uncharacterized LOC105283366	LOC105282680	glucosylceramidase
26	LOC105279559	glycine receptor subunit alpha-2-like	LOC105276699	uncharacterized LOC105276699	LOC105277551	glycine N-methyltransferase	LOC105281428_Q	queen Vg
27	LOC105287812	uncharacterized LOC105287812	LOC105275435	alpha-(1,3)-fucosyltransferase 6	LOC105287207	kinesin-like protein Klp61F	LOC105281103	pancreatic triacylglycerol lipase-like
28	LOC105281784	transcription termination factor 2	LOC105279012	uncharacterized LOC105279012	LOC105287679	kinetochore protein NDC80 homolog	LOC105277939	uncharacterized LOC105277939
29	LOC105278931	venom allergen 3	LOC105278924	uncharacterized LOC105278924	LOC105274638	kinesin-like protein KIF18A	LOC105275854	annulin
30	LOC105277551	glycine N-methyltransferase	LOC105275769	uncharacterized LOC105275769	LOC105284779	uncharacterized LOC105284779	LOC105287317	actin, muscle-like
31	LOC105285597	uncharacterized LOC105285597	LOC105287317	actin, muscle-like	LOC105284181	peptidyl-prolyl cis-trans isomerase D	LOC105281449	muscle M-line assembly protein unc-89-like
32	LOC105283621	synaptic vesicle glycoprotein 2C	LOC105277469	tubulin beta chain	LOC105287093	uncharacterized LOC105287093	LOC105284373	myosin light chain alkali
33	LOC105279939	lymphokine-activated killer T-cell-originated protein kinase	LOC105275469	sarcalumenin	LOC105276433	box A-binding factor	LOC105281784	transcription termination factor 2
34	LOC105283366	uncharacterized LOC105283366	LOC105287714	protein hunchback	LOC105279939	lymphokine-activated killer T-cell-originated protein kinase	LOC105281010	uncharacterized LOC105281010
35	LOC105281245	uncharacterized LOC105281245	LOC105284373	myosin light chain alkali	LOC105278724	fatty acid synthase-like	LOC105280202	glycine-rich cell wall structural protein
36	LOC105275821	dnaJ homolog subfamily C member 22	LOC105281448	twitchin	LOC105287758	uncharacterized LOC105287758	LOC105285169	sperm flagellar protein 2
37	LOC105281786	hyaluronan mediated motility receptor	LOC105284497	intraflagellar transport protein 46 homolog	LOC105275039	uncharacterized LOC105275039	LOC105277551	glycine N-methyltransferase
38	LOC105283233	fibrillin-1	LOC105281212	protein rhomboid	LOC105280537	uncharacterized LOC105280537	LOC105277439	ADP-ribosylation factor-like protein 6
39	LOC105277056	neuroparsin-A	LOC105284074	phospholipase B1, membrane-associated	LOC105279590	uncharacterized LOC105279590	LOC105275469	sarcalumenin
40	LOC105279064	uncharacterized LOC105279064	LOC105281920	aminopeptidase N-like	LOC105281082	uncharacterized LOC105281082	LOC105287204	twitchin
