# Supplementary material for: Clonal raider ant brain transcriptomics identifies candidate molecular mechanisms for reproductive division of labor
Source: BMC Biol. 2018 Aug 13;16:89. doi: 10.1186/s12915-018-0558-8 (PMC6090591; doi:10.1186/s12915-018-0558-8)
Supplement: Supplementary file 10 — Summary of clusters enriched for differentially expressed genes (DEGs) and transcription factor binding sites. (PDF 53 kb) [file 12915_2018_558_MOESM10_ESM.pdf]

Summary of clusters enriched for differentially expressed genes (DEGs) and transcription factor binding sites (TFBSs).

Cluster #	Time interval with maximal change in expression	Maximum change vector	Number of genes in the cluster	Number of DEGs in the cluster	TFBSs in the cluster
Reproduction to brood care					
8	12-24 hrs	-0.35	134	48	*fkh*
93	0-12 hrs	0.25	134	16	*Mad, usp, opa, btd, h, brk*
90	24-48 hrs	0.16	151	30	*Mad, usp,opa, btd, h, hkb*
70	24-48 hrs	0.22	162	45	*Mad, usp, opa, Trl*
Brood care to reproduction					
19	48-96 hrs	0.59	245	58	*fkh*
21	48-96 hrs	-0.28	315	25	*Mad, usp, opa, btd, Trl, h, brk, gcm2, dl, Ets21C*
4	48-96 hrs	1.16	156	31	*ap, Awh, br, btn, C15, CG11617, CG18599, CG32105, CG32532, CG4328-RA, CG9876, Dbx, Dll, ems, en, eve, exex, fkh, gt, H2.0, hbn, HGTX, inv, lab, lbl, Lim1, Lim3, lms, NK7.1, onecut, otp, pb, PHDP, Pph13, repo, ro, Rx, Scr, Six4, slou, slp1, unpg, Vsx1, Vsx2, zen, zen2*
35	0-12 hrs	-0.57	167	19	*al, Awh, CG11294, CG32532, CG9876, Dr, en, exex, ftz, hbn, ind, inv, Lim1, OdsH, otp, Pph13, repo, ro, Rx, slou, unc-4, unpg, Vsx1, Vsx2*

Maximum change vector: magnitude and direction of the largest average gene expression change between time intervals. TFBS classes identified are as follows: Basic leucine zipper factors (bZIP) – gt; C2H2 zinc finger factors – br, btd, hkb, opa, Trl, opa; Fork head / winged helix factors – fkh, slp1; GCM domain factors – gcm2; Homeo domain factors – al, ap, Awh, btn, C15, CG11294, CG11617, CG18599, CG32105, CG32532, CG4328-RA, CG9876, Dbx, Dll, Dr, ems, en, eve, exex, ftz, H2.0, hbn, HGTX, ind, inv, lab, lbl, Lim1, Lim3, lms, NK7.1, OdsH, onecut, otp, pb, PHDP, Pph13, repo, ro, Rx, Scr, Six4, slou, unc-4, unpg, Vsx1, Vsx2, zen, zen2; Nuclear receptors with C4 zinc fingers – usp; Rel homology region (RHR) factors – dl; SMAD/NF-1 DNA-binding domain factors – Mad; Zipper-type – h; Tryptophan cluster factors – Ets21C.
