# Supplementary material for: Clonal raider ant brain transcriptomics identifies candidate molecular mechanisms for reproductive division of labor
Source: BMC Biol. 2018 Aug 13;16:89. doi: 10.1186/s12915-018-0558-8 (PMC6090591; doi:10.1186/s12915-018-0558-8)
Supplement: Supplementary file 12 — Number of replicates in the analyses (after outlier removal). (PDF 28 kb) [file 12915_2018_558_MOESM12_ESM.pdf]

Number of replicates in the analyses (after outlier removal).

Transition	BR transition					RB transition				
Time point	Ctl	12h	24h	48h	96	Ctl	12h	24h	48h	96h
Number of replicates	**4**	**8**	**8**	**8**	**8**	**4**	**8**	**8**	**8**	**8**
